# Natural experiments for the evaluation of place-based public health interventions: a methodology scoping review

**DOI:** 10.3389/fpubh.2023.1192055

**Published:** 2023-06-22

**Authors:** Patricia N. Albers, Chiara Rinaldi, Heather Brown, Kate E. Mason, Katrina d'Apice, Elizabeth McGill, Cheryl McQuire, Peter Craig, Anthony A. Laverty, Morgan Beeson, Mhairi Campbell, Matt Egan, Marcia Gibson, Maxwell Fuller, Amy Dillon, David Taylor-Robinson, Russell Jago, Kate Tilling, Benjamin Barr, Falko F. Sniehotta, Matthew Hickman, Christopher J. Millett, Frank de Vocht

**Affiliations:** ^1^Population Health Sciences, Bristol Medical School, University of Bristol, Bristol, United Kingdom; ^2^Department of Public Health, Environments and Society, London School of Hygiene and Tropical Medicine, London, United Kingdom; ^3^Health Research, Lancaster University, Lancaster, United Kingdom; ^4^Department of Public Health Policy and Systems, University of Liverpool, Liverpool, United Kingdom; ^5^Centre for Health Policy, University of Melbourne, Parkville, VIC, Australia; ^6^MRC/CSO Social and Public Health Sciences Unit, School of Health and Wellbeing, University of Glasgow, Glasgow, United Kingdom; ^7^School of Public Health, Imperial College London, London, United Kingdom; ^8^Newcastle University Business School, Newcastle University, Newcastle upon Tyne, United Kingdom; ^9^Department of Public Health, Policy and Systems. University of Liverpool, Liverpool, United Kingdom; ^10^The National Institute for Health Research, Applied Research Collaboration West (NIHR ARC West), University Hospitals Bristol and Weston NHS Foundation Trust, Bristol, United Kingdom; ^11^Centre for Exercise, Nutrition and Health Sciences, School for Policy Studies, University of Bristol, Bristol, United Kingdom; ^12^MRC Integrative Epidemiology Unit, University of Bristol, Bristol, United Kingdom; ^13^Institute of Population Health, University of Liverpool, Liverpool, United Kingdom; ^14^NIHR Policy Research Unit Behavioural Science, Newcastle University, Newcastle upon Tyne, United Kingdom; ^15^Department of Public Health, Social and Preventive Medicine, Medical Faculty Mannheim, Heidelberg University, Heidelberg, Germany

**Keywords:** public health, public health policy, natural experiments, quasi experiments, evaluations, place-based

## Abstract

**Introduction:**

Place-based public health evaluations are increasingly making use of natural experiments. This scoping review aimed to provide an overview of the design and use of natural experiment evaluations (NEEs), and an assessment of the plausibility of the *as-if* randomization assumption.

**Methods:**

A systematic search of three bibliographic databases (Pubmed, Web of Science and Ovid-Medline) was conducted in January 2020 to capture publications that reported a natural experiment of a place-based public health intervention or outcome. For each, study design elements were extracted. An additional evaluation of *as-if* randomization was conducted by 12 of this paper's authors who evaluated the same set of 20 randomly selected studies and assessed ‘*as-if* ' randomization for each.

**Results:**

366 NEE studies of place-based public health interventions were identified. The most commonly used NEE approach was a Difference-in-Differences study design (25%), followed by before-after studies (23%) and regression analysis studies. 42% of NEEs had likely or probable *as-if* randomization of exposure (the intervention), while for 25% this was implausible. An inter-rater agreement exercise indicated poor reliability of *as-if* randomization assignment. Only about half of NEEs reported some form of sensitivity or falsification analysis to support inferences.

**Conclusion:**

NEEs are conducted using many different designs and statistical methods and encompass various definitions of a natural experiment, while it is questionable whether all evaluations reported as natural experiments should be considered as such. The likelihood of *as-if* randomization should be specifically reported, and primary analyses should be supported by sensitivity analyses and/or falsification tests. Transparent reporting of NEE designs and evaluation methods will contribute to the optimum use of place-based NEEs.

## 1. Introduction

“Place-based public health interventions” can be described as activities, programmes, or policies delivered at a local, regional, or national level, that aim to improve health or reduce health inequalities of the community. “Place” is the study of space or places, and in health often specifically refers to the places where people are born, grow, live, work, and age (1). It is well established that contextual factors such as “place” play a role in creating and perpetuating health inequities (2). Moreover, place-based interventions are an important policy lever for influencing the wider determinants of health (3). Community and place-based public health interventions are therefore common: a recent umbrella review found 13 systematic reviews, covering 51 unique studies (published since 2008) looking at the effectiveness of place-based interventions to improve public health and reduce health inequalities (4). One of the most famous examples of a place-based natural experiment in public health is that of John Snow and the Broad Street pump, where he showed that people obtaining water from that pump were being infected by cholera (5). Other well-known examples of place-based natural experiments at the national level are the evaluation of the effects of the “Dutch Hunger Winter” at the end of World War 2, in which food was scarce in the occupied West of the Netherlands, but not in the liberated South (6); the effects of the US blockade of Cuba following the collapse of the Soviet Union (7); and the impact of a drastic improvement in air pollution during the Beijing Olympics as a result of various place-based interventions such as reducing traffic (8). These examples all demonstrate how the spatial location, or place, as the target of an intervention can positively impact on population health. A recent local level example is the evaluation of the impact of Transport for London's ban of fast food advertising on household purchases (9). The missed opportunities [where place-based interventions were implemented but could not be evaluated by a randomized controlled trial (RCT)] have resulted in a lack of evidence-based policy, with many public health interventions only being supported by associative evidence from observational studies (10). Indeed, for the above examples, as well as for many other evaluations of place-based public health interventions, it is not possible to conduct the traditionally preferred method of an RCT. RCTs have the distinct advantage for causal inference that, when properly carried out, randomization of exposure allocation minimizes or eliminates residual confounding of the exposure-outcome association. Causal inference, often in the absence of an RCT, aims to establish whether an exposure or intervention is an independent predictor of an outcome, going beyond simply exploring associations (11). However, whereas RCTs are traditionally considered the “gold standard” in medicine due to their (at least theoretically) high internal validity, in other fields RCTs are less favored due to low external validity.

For many (place-based) policy interventions therefore, there remains the opportunity for evaluation using quasi-experimental or natural experimental designs, which we refer to collectively as natural experiment evaluations (NEEs). Although the terms quasi and natural experiments are often used interchangeably rchangeable, a stricter view of the two can be used, whereby in quasi-experiments there is no assumption of randomization or *as-if* randomization, while natural experiments take advantage of random or *as-if* random assignment (12). The interest of public health in NEEs is relatively recent, but in cognate fields (such as economics) the use and development of NEE methodology has been much more pronounced (10). NEEs can have additional advantages over RCTs for the evaluation of place-based public health interventions in that interventions may have already been implemented or are to be implemented by, for example, local councils, making it unethical or impractical to randomize. Additionally, the cost and scale of an RCT may be prohibitive. The value that NEEs have in the evaluation of public health policies, especially where they can be aligned to the policy cycle, has resulted in their increased use to inform public health policy (13).

NEEs combine features of (i) RCTs, in which an event or process can be used to divide the population into exposed and unexposed groups (or different levels of exposure), and (ii) observational studies, as the allocation of participants to a study group is not under the control of the researcher. The former, at least in theory, makes NEEs less susceptible to confounding than many other observational designs (14, 15). Although there are a few variations in the definition of a natural experiment, an important commonality is that the exposure or intervention (and as such group allocation) are not controlled by the researcher (14, 16). This lack of control over exposure assignment may complicate attempts to draw causal inferences, as it may be more difficult to determine whether the allocation of individuals or groups to intervention or control group(s) is truly independent of the outcome (14, 16). To improve the strength of causal statements from NEEs, Dunning (12) proposed a stricter definition of NEEs in which a study is only considered an NEE if the allocation of exposure is *as-if* random. *The as-if random assumption* posits that in some circumstances where exposure allocation has not been controlled by the researchers, allocation can be considered to originate from a random process, thereby replicating randomization in an experiment (with studies having implausible *as-if* randomization categorized as quasi experiments) (12). A classic example of this is the US draft lottery which took place during the Vietnam War where men born between certain periods were assigned, at random, a lottery number based on their date of birth. All men with birth dates corresponding to the first 195 numbers were called to service, a similar process ran three times during the war period (12, 17). The *as-if* random allocation of exposure however, is not easily, if ever, verifiable (16, 18), which has important implications for the extent to which causal claims can be made (14).

In its guidance on the use of natural experiments (13), the UK Medical Research Council (MRC) does not recommend particular designs or analytic methods for NEEs. In practice, the evaluation of NEEs is typically conducted using several different evaluation designs, including post-intervention comparison studies, pre-post studies, difference-in-differences (DiD) studies, regression discontinuity studies, or (controlled) interrupted time series studies (19), and various different statistical methods to analyse these, including regression adjustments, multilevel methods, propensity score matching or weighting, synthetic controls or the use of instrumental variables (19). For illustration, in a recent review exploring the use of NEEs to improve public health evidence for obesity, 32% of included studies were pre-post longitudinal studies with a comparison group, and four (8.5%) were interrupted time series studies, with the remainder using mostly descriptive methods such as repeated cross-sectional studies, post-test observational studies only, and longitudinal pre/post single group cohort studies (20).

To support causal inferences NEEs can benefit from additional sensitivity and falsification analyses, such as temporal falsification methods where intervention implementation times are adjusted, or spatial falsification methods using different groups (e.g., different geographical areas) regardless of the exposure, and/or by conducting methodological triangulation of using different statistical analysis (14). Negative controls, where no impact from the intervention is expected, are also a valuable tool for identifying and correcting bias (21).

Because of the increasing importance of NEEs in the context of the evaluation of (placed-based) public health interventions, it is important to evaluate the methodological strength on which this evidence of effectiveness is based. We aimed to explore the practices and methods used, trends and developments in the use of methods and concepts, their strength for causal inference, and provide recommendations for addressing the main weaknesses in published place-based NEEs in public health.

## 2. Materials and methods

Scoping reviews are typically chosen when the purpose of the review is to explore gaps in the subject matter, understand the extent of a body of literature, explain theories or constructs, or examine specific research practices (22, 23). Scoping reviews are a process of summarizing a body of evidence (24), but with key differences to systematic reviews that the quality of the included papers is not normally assessed formally (24) while also no quantitative synthesis was conducted. The current scoping review aims to understand the extent of the body of literature on place-based NEEs in public health, examine specific research practices, and provide recommendations for future improvements of such evaluations.

A systematic search of three bibliographic databases (Pubmed, Web of Science, and Ovid-Medline) was conducted in January 2020 to capture publications that reported a natural experiment of a place-based public health intervention or outcome. The search strategy used a modified version of the one used in a similar study on the use of complex systems in place-based public health (25), but systems-level terms were replaced with natural experiment terms. Three separate searches using similar terms were conducted [details provided in Online Supplement material (OSM) Supplementary Table S1]. Search 1 included natural experiment and public health terms. Search 2 included natural experiment and evaluation and public health terms, in order to identify additional studies classified by evaluation specific items not covered in Search 1. Search 3 similarly aimed to identify additional papers missed by Searches 1 and 2 by including public health terms with additional terms related to common study design and analytic methods used in NEEs. All three searches were run on all three databases, where titles and abstracts where searched. Due to resource limitations we limited the scoping review to studies published in the English language. For this review we defined public health as efforts to prevent illness, increase life expectancy, improve health and address health inequalities. Results were merged and duplicates removed before being added to Rayyan (https://www.rayyan.ai/).

Titles and abstracts were screened and marked for in/exclusion by a minimum of two reviewers. In the event of a difference of opinion a third reviewer made the final decision. Papers were excluded if the title or abstract included clinical and not public health outcomes, were based on Mendelian randomization, or if the paper was a review, protocol, policy debate, or discussion piece. A study was included if it met the following eligibility criteria:

- Study self-identified as being an NEE, regardless of the actual methods use, allowing us to explore the scope of self-defined NEEs, and the predominant practices among them.
- Study evaluated a place-based public health intervention. We defined this as activities, programmes, or policies delivered at a local, regional, or national level, that aimed to improve health or reduce health inequalities of the community. Examples of such interventions include a policy to restrict new fast-food outlets near schools or if the density surpasses a certain threshold of retail outlets (26), or other changes to a community, or policy changes, for instance a change in policy on access to emergency contraception (27).
- Study reported an intervention which addressed a public health relevant topic. Some examples of health relevant topics include; substance use, physical activity, weight, nutrition, and mental health. We excluded clinical outcomes and papers using Mendelian randomization as the latter are typically not conducted as part of place-based public health policy evaluation.
- Study needed to report empirical findings rather than hypothetical scenarios. Therefore reviews, protocols, policy debates, or discussion pieces were also not included.

Studies from any country were eligible, however, we limited the search to studies published in the English language.

A data extraction template was populated in Microsoft Excel and tested by PA and FdV using four papers, after agreement about the process, the remaining extraction was conducted using the final template. One member of the study (PNA, FdV, CR, HB, KEM, KD, EM, CM, PC, AAL, MB, MC, ME, MG, MF, AD, or DTR) team extracted an allocation of papers after which PA checked the completeness of the extractions and in places where the extracted data were incomplete or the author was unsure, PA completed these.

The extraction template with an explanation of what was collected in each field is provided in Table 1.

**Table 1:** Extraction template with details of what was collected in the different fields.

**Extraction template**	***Details***
**Reference details**	
Reference number	-
Title	-
url	-
Authors	-
Year	-
Funder	-
**Study characteristics**	
Population	Open ended: captured details such as ‘Children born in X region of X Country'
Evaluation	National policy
	Local policy
	Other (Open ended)
Aim	Generic health improvement
	Wellbeing
	Nutrition
	Weight
	Substance misuse
	Mental health
	Fitness
	Other (Open ended)
**Methodological characteristics**	
Data type	Individual level
	Aggregated data
Approach	Instrumental variable
	Synthetic control
	Difference-in-differences
	Interrupted timeseries
	Before-after comparison
	After comparison
	Other (Open ended)
As-if randomisation	Implausible
	Possible
	Probably
	Likely
Comparator	Open ended: captured details such as ‘control groups or unexposed residents'
Sensitivity or falsification	No
	Temporal (different timepoints)
	Spatial (different cases/controls)
	Both
	Other (Open ended)
Statistical methodology (further details)	Open ended: captured details such as ‘Exploratory analyses compared pollution and health'
**Outcomes**	
Outcome measured	Open ended
Effect size/Results	Open ended
Confidence/Credible interval	Open ended
**Notes**	Open ended

This template collected the following data; *Year, Population, Intervention/Exposure evaluated, Individual/Aggregated data, As-if randomization* (Implausible/Possible/Probably/Likely), *Approach* (Instrumental variable/Synthetic control/Difference-in-differences (DiD)/Interrupted time series/Before-after comparison/After comparison/Other), *Comparator, Definition of comparator, Sensitivity/Falsification* (No/Temporal (different timepoints)/Spatial (different cases/controls)/Both/Other), *Evaluation* (Local policy/National policy/Other), and *Aim* (Physical activity/Miscellaneous outcomes/Mental health/Nutrition/Substance use/Weight/Well-being/Other). “Miscellaneous outcomes” was provided as a category for studies that could not easily be categorized or did not address a specific single outcome, and included for instance “hospital admissions” or “influenza prevention”. A brief description of the various approaches is provided below [more details are available in for example (19)]:

- Instrumental variable: using a variable that is only associated with the exposure and outcome and no other confounders or unmeasured error, these variables can often replicate randomization.
- Synthetic control: allows a counterfactual to be constructed based on a weighted average of the outcome from a group similar to the exposed.
- Difference-in-differences: explores changes in the outcome from pre to post between the exposed and unexposed group but taking account of the trend of the unexposed group.
- Interrupted time series: chronological sequence of outcome data which is then interrupted by the exposure or intervention, the trend after the interruption is compared to the counterfactual.
- Before- after comparison: measuring the outcome in a group of participants before and after an intervention.
- After comparison: comparison of outcomes in an exposed and unexposed group.

We collected additional data on the statistical methodology using an open field for further details. Finally, we included a field for any additional notes of relevance not covered by the pre-specified categories. For some sections on the template the categories were not mutually exclusive, however, those carrying out the extraction selected the category the best aligned with how the author/s of that individual paper described their own study. For most sections we provided an “*Other*” category intended for use when a reviewer felt the study did not match one of the provided categories. We also provided an open-ended text field where the reviewer could write the details for the particular study. For example, if a reviewer believed the study was a time series study rather than an interrupted time series study.

The *Population, Intervention/Exposure evaluated, Comparator*, and *Definition of comparator* fields, as well as all the “*Othe*r” fields were all open-ended. These fields were manually coded into groups and themes that directly corresponded to the topics. Where a field did not completely match an existing code a new code was created. After coding, all the new codes were compared and where the underlying theme was the same, these were merged. When one of these open-ended fields were left blank they were coded as missing. Using the *Population* field, we coded the countries from where the studies reported data.

For the comparator and definition of comparator fields the open-ended responses were used to create a coded variable which included Control groups/Control areas/Pre-intervention (same population)/Temporal controls (different population and different time points)/Matched controls/Synthetic controls or counterfactuals/Sibling controls or control products/No comparator. Where noted, we used the terminology stated by the authors. Control groups refers to comparison groups that were not specifically matched, they were a selection of other (possibly related) groups, for example schools in the same region. Control areas refers to a selection of individuals from a particular area which was different to the primary study area, for example different countries. Pre-intervention controls refers to data from the same sample of individual before the intervention began. Temporal controls refers to a sample of people before the intervention, however, not necessarily the same sample of people. For example adult respondents of a survey before a particular event, compare to an adult sample of respondents to the same survey but after the event. Matched controls refers to controls where the authors specifically stated that they were matched on various criteria. Counterfactuals refers to controls whereby the study authors specifically explained the creation of a counterfactual based on pool of various control options. No comparator refers to studies that did not include any comparator or studies where authors compared groups of people with different levels of exposure.

The methodology used was categorized based on how the authors of each study described their approach (Instrumental variable/Synthetic control/DiD/Interrupted time series/Before-after comparison/After comparison/Other), and was as a result an amalgamation of designs and statistical methods. Studies that did not use one of these approaches were categorized as “Other”. Two studies defined themselves as before-after studies and were subsequently coded as DiD studies, because despite the authors referring to these as before-after studies, they in fact describe a DiD study.

The plausibility of *as-if* randomization in each study was judged by the reviewers who carried out the extraction. In the data extraction template plausibility was categorized in four alternatives (Implausible/Possible/Probably/Likely). *As-if* randomization was assessed by each reviewer independently, where this was not provided by the study authors. In some situations where reviewers were uncertain an additional reviewer (PA) independently assessed this. There are currently no specific criteria or thresholds to establish “*as-if* ” randomization of exposure; therefore, we relied on the traditional methods used to assess the strength of observational studies to establish causality, such as possible exposure (self)selection and other risks of bias (16). These often have to be inferred from manuscripts rather than that they are explicitly stated by the authors, leaving room for subjective assessments. One of the key considerations we used to assess *as-if* randomization was that participants did not have the capacity, information, or incentive to self-select into a study group. And similarly policy makers or other individuals assigning participants to groups also did not have the capacity, information, or incentive to assign individuals to a particular group.

Given that *as-if*-random is an essential criterion impacting on the strength of any causal conclusions (28), we conducted an additional exploratory exercise. Twelve of this paper's authors evaluated the same set of 20 natural experiments, randomly selected from the list of identified studies, and assessed “*as-if* ” randomization for each of them using the same categories used in the extractions template (Implausible/Possible/Probably/Likely). Intra-class correlations were calculated to determine inter-rater agreement using the R *irr* package based on two-way random effects where absolute agreement is important for single raters (29).

## 3. Results

**Figure 1:**
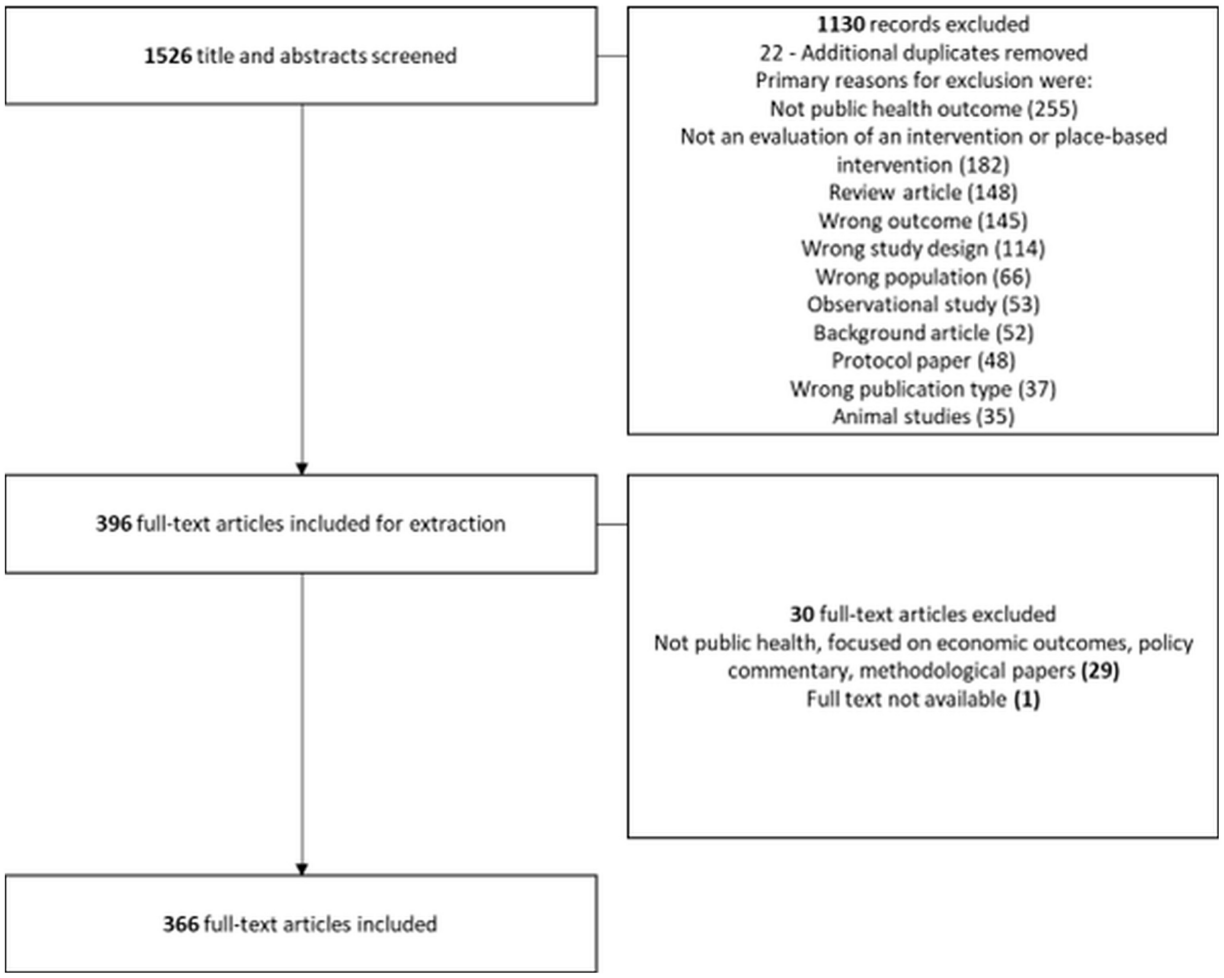
PRISMA Flow Diagram. Note that the total number of excluded studies per categories does not equal the total number of those excluded because studies may have been assigned more than one label.

A total of 1,526 titles and abstracts were screened, resulting in 396 studies included for data extraction. An additional 29 studies were later excluded at the data extraction stage primarily because they were not related to public health or were a policy commentary or methodological paper. One further study was excluded because the full-text was not available, resulting in a total of 366 papers included for extraction. The flow diagram of the literature search is depicted in Figure 1, the total number of excluded studies per categories does not equal the total number of those excluded because studies may have been assigned more than one label. A table with all included references is provided in OSM Supplementary Table S2.

**Figure 2:**
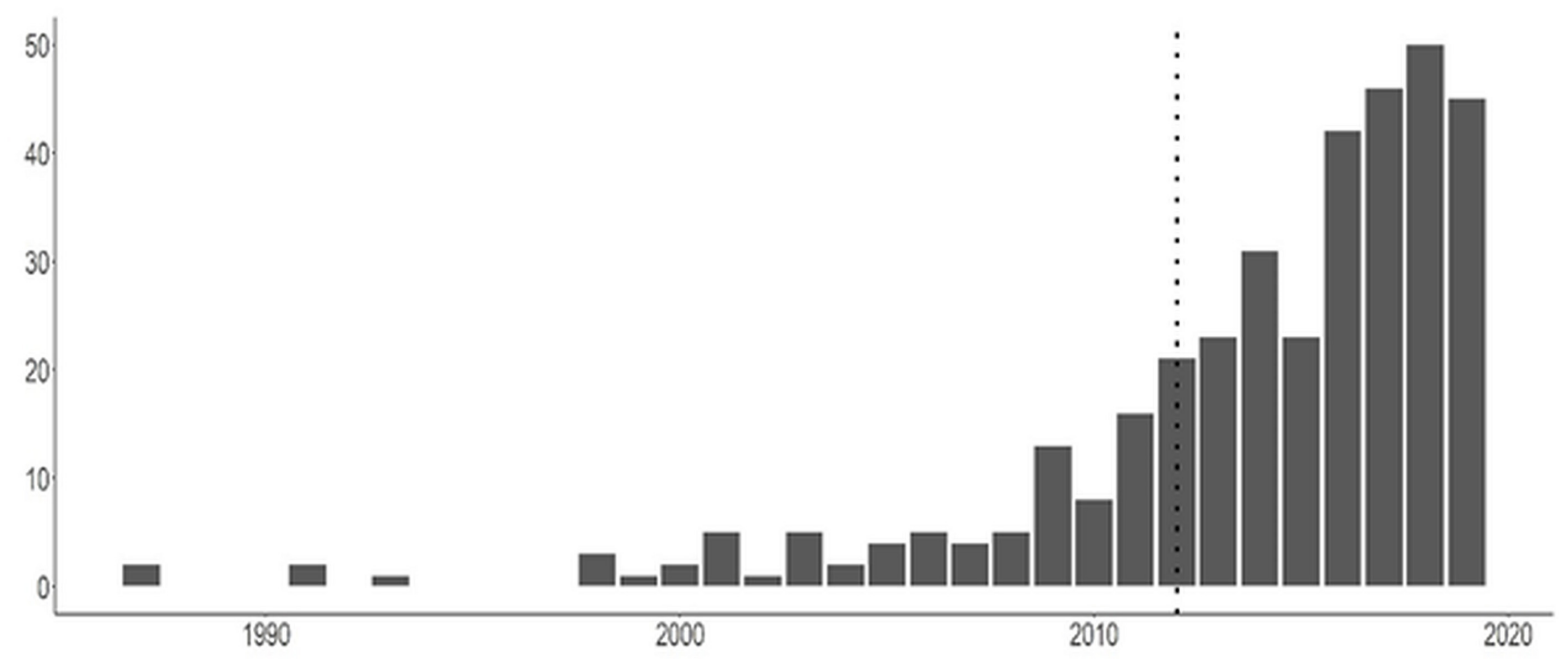
Number of studies identified as “natural experiment evaluations” by their authors up to December 2019 (six studies from early 2020 included in this study, but not shown in this Figure to avoid erroneous inferences on trends). Dottedline indicates publication MRC guidance were published (16).

The annual numbers of published place-based NEEs of public health interventions from 1987 to 2019 are shown in Figure 2 and indicate a pattern of increase in their use from about 2009 onwards. Our search was run in early January 2020, six studies were picked up from 2020 but are not show in the plot below. For reference, the publication of the MRC guidance in 2012 (16) is shown by the dotted line in the figure.

Included studies were from 54 mostly English-speaking countries (Table 2). One third of studies originated from the USA (33.3%, *n* = 122), followed by the UK (13%), Australia (7%) and Canada (6%). In the USA, studies were predominantly DiD studies, while in the UK, Australia, and Canada studies used predominantly before-after comparison study designs.

**Table 2:** Study characteristics of the included studies.

**Study characteristics**	***n* (%)**
**Country**	
USA	122 (33.3)
UK	48 (13.1)
Australia	26 (7.1)
Canada	22 (6.0)
Sweden	15 (4.1)
Multiple	14 (3.8)
China	12 (3.3)
Denmark	10 (2.7)
Finland	9 (2.5)
France	5 (1.4)
Norway	5 (1.4)
Other	60 (16.4)
**Evaluation type**	
Local policy	123 (33.6)
National policy	122 (33.3)
Other	121 (33.1)
Neighborhood interventions	38 (32.8)
Nutrition and diet interventions	15 (12.9)
Schooling or school policy	9 (7.8)
Healthcare change	8 (6.9)
Economic change	7 (6.0)
Natural or manmade disasters	4 (3.4)
**Aims of included studies**	
Substance use	68 (18.8)
Miscellaneous outcomes	57 (15.7)
Physical activity	48 (13.3)
Nutrition	32 (8.8)
Weight	22 (6.1)
Mental health	16 (4.4)
Well-being	11 (3.0)
Other	45 (12.4)
Pregnancy related	32 (8.8)
Access to healthcare	11 (3.0)
Mortality	10 (2.8)
Crime	10 (2.8)

### 3.1. Study characteristics

Nearly 67% (245) of included studies investigated the effect of a policy (national or local), while the remaining third were classified as evaluations of community initiatives. The two most frequently described type of evaluation explored the impact of neighborhood infrastructure changes (33%), such as the addition of outlets or green space, and nutrition and diet interventions (13%). These were followed by schooling/school policy or school environment changes (8%), health care changes (7%), and economic changes (6%) (see Table 2).

The specific aims of the studies were categorized as seeking to evaluate the impact of a policy or other change on physical activity, miscellaneous outcomes, mental health, nutrition, substance use, weight, wellbeing, or other. Substance use (18.8%) and physical activity (13.3%) were the primary areas of research, with various miscellaneous outcomes interventions evaluated in 15.7% of included studies. A high proportion (30.1%, *n* = 110) of studies did not align with the prelisted categories and from this four additional categories were identified. These included the evaluation of policies or other interventions aimed at impacting primarily pregnancy-related outcomes (8.7%), access to healthcare (3%), crime (2.7%), and mortality (2.7%).

### 3.2. Methodological characteristics

An overview of the methodological characteristics of the included studies is provided in Table 3. Most NEE studies (68%, *n* = 248) were based on individual-level data for their primary analyses, with the remaining 32% based on aggregate data such as counts of incident cases.

**Table 3:** Methodological characteristics of the included studies.

**Methodological characteristics**	***n* (%)**
**Data type**	
Aggregate	248 (67.8)
Individual	118 (32.2)
**Approach**	
Instrumental variable	22 (6.0)
Synthetic control	6 (1.6)
Difference-in-differences (DiD)	93 (25.4)
Interrupted time series	40 (10.9)
Before-after comparison	84 (23)
After comparison	18 (4.9)
Other	103 (28.1)
Regression analysis	71 (68.9)
Time series analysis	13 (12.6)
Other Econometric methods	3 (2.9)
Descriptive analysis	2 (1.9)
Other	8 (7.7)
Missing	6 (5.8)
**Comparators**	
Comparison groups	124 (33.9)
Comparison areas	92 (25.1)
Pre-intervention	57 (15.6)
No comparator	50 (13.7)
Temporal control	26 (7.1)
Matched controls	7 (1.9)
Within sample	3 (0.8)
Synthetic control or counterfactuals	3 (0.8)
Sibling	4 (1)
***As-if*** **Randomisation**	
Likely	69 (19.1)
Probable	84 (23.2)
Possible	109 (30.1)
Implausible	90 (24.9)
Cannot tell	10 (2.8)

The most commonly used NEE approach was the DiD study design (25%, *n* = 93). This was followed by before-after comparison studies (23%, *n* = 84) and interrupted time series studies (11%, *n* = 40). Statistical approaches that have become more frequently used included instrumental variables (used in 6% of NEEs) and synthetic controls (used in 1.6%). 28% of studies recorded something other than these approaches, with 68.9% of these reporting regression-based analysis studies where no specific change in exposure is modeled and 12.6% reporting time series analysis, to model variation in exposure but without an element of the specific analysis of a change in exposure (14). The remaining studies used “other econometric methods” (2.9%) or descriptive analysis (1.9%). Only one study reported using propensity score matching, captured under “other”.

Table 3 shows most studies included a comparator either in the form of control groups (34%), control areas (25%), or used the same population before the intervention (16%). 13% of studies had no comparison group at all, and only three studies (<1%) used synthetic control or formal counterfactuals.

42.3% of all included studies were assessed as having likely (19.1%) or probable (23.2%) *as-if* randomization, while as many as a quarter (24.9%) of NEEs were assessed as having ‘implausible *as-if* randomisation'. 30% of NEEs were classified as ‘possible *as-if* randomisation'. A small proportion of studies (2.8%) provided insufficient details for evaluation and were coded as “Unsure or cannot tell”. The majority of studies with likely *as-if* randomization were evaluating national policy (44%), with local policy evaluations being the least likely to be categorized as having likely *as-if* randomization (18%). There was no obvious pattern between country of publication and plausibility of *as-if* randomization in study design.

Table 4 shows the *as-if*-random classification for each of the study types. NEEs based on synthetic controls (*n* = 6) were assessed as having the highest proportion of probable or likely *as-if* randomization (83.3%) followed by instrumental variable studies (*n* = 22; 50.0%) and DiD studies (*n* = 93; 47.4%). Other methods and “after comparisons” (*n* = 18) included about 44% of studies with plausible or likely *as-if* randomization, while for interrupted time series studies and before-after comparison studies only about 32% had plausible or likely *as-if* randomization. On the other hand, 33% of “after comparison” studies and about 30% of “other” studies, interrupted time series studies, and before-after comparison studies were assessed as having implausible *as-if* randomization.

**Table 4:** As-if randomisation by different statistical or design approach.

	***Implausible***	***Possible***	***Probable***	***Likely***	**Cannot tell**	***Total***
	***n (%)***	***n (%)***	***n (%)***	***n (%)***	***n (%)***	***N***
Synthetic control	1 (16.7)	0 (0)	3 (50.0)	2 (33.3)	0 (0)	6
Instrumental variable	3 (13.6)	8 (36.4)	5 (22.7)	6 (27.3)	0 (0)	22
Difference-in-differences (DiD)	14 (15.1)	33 (35.5)	26 (28.0)	18 (19.4)	2 (2.2)	93
Other	30 (29.1)	25 (24.3)	24 (23.3)	22 (21.4)	2 (1.9)	103
After comparison	6 (33.3)	4 (22.2)	5 (27.8)	3 (16.7)	0 (0)	18
Interrupted time series	12 (30.0)	12 (30.0)	6 (15.0)	7 (17.5)	3 (7.5)	40
Before-after comparison	24 (28.6)	30 (35.7)	16 (19.0)	11 (13.1)	3 (3.6)	84

Given that there are no specific criteria for assessing *as-if* randomization we specifically set out to assess inter-rater agreement for *as-if* randomization as this is a subjective classification that has nonetheless important implications for causal inference. *As-if* randomization was not reported in the studies included in the exercise. The validation exercise was completed for 18 of the 20 randomly selected studies by 10 assessors (there were incomplete responses for two paper and two assessors, which were excluded from the analysis). The corresponding intraclass correlation coefficient (ICC; 95% confidence interval) was 0.35 (0.19–0.47) indicating poor reliability. Complete agreement on the 5-point scale across all ten authors did not occur, with agreement within one unit occurring for only 6% of studies (details in of all individual assessments are provided in OSM Table 3). As such it is worth noting the results presented in Table 4 are based on subjective assessments made by different authors of this paper.

Half (50%) of evaluations reported some form of sensitivity or falsification analysis. The most frequently used method was spatial falsification (17%), in which different control groups, generally geographical areas where an intervention was not implemented, are included as intervention areas (to assess whether any effect is unique to the intervention area). 10% of studies included a temporal falsification analysis in which alternate time points for the intervention were included (to assess whether any effect was unique to the intervention timepoint) (14). Some studies reported using both spatial and temporal falsification analyses (8%). In addition, 16% of NEEs reported “other” methods, which was mainly the redefinition of model variables, adding additional variables to their models, or changes in the model specifications.

Sensitivity and falsification analyses stratified by statistical approach are shown in Table 5. Within the categories, the proportion of studies that did not include any falsification or sensitivity analyses varied from 17% to 55%. In contrast, although only six studies were included, 50% of NEEs using synthetic control methods used both temporal and spatial methods. Of the instrumental variable studies, 18% (*n* = 4) reported using redefining variables in the models as a form of sensitivity analysis. For DiD studies about one in four studies (24 of 93) used spatial methods. For interrupted time series studies nearly equal proportions used temporal (12.5%, *n* = 5), spatial (15%, *n* = 6) or both (15%, *n* = 6). Before-after comparison studies, after comparison studies, and “other” studies had the lowest proportions of additional sensitivity or falsification analyses to strengthen causal inference; 30%, 33%, and 45%, respectively.

**Table 5:** Percentage of sensitivity or falsification approaches used with different statistical approaches.

	**No sensitivity or falsification n(%)**	**Spatial n(%)**	**Temporal n(%)**	**Both n(%)**	**Redefined variables n(%)**	**Aggregated variables n(%)**	**Model specification n(%)**	**Other n(%)**	**Total n**
Synthetic control	1 (16.67)	1 (16.67)	0 (0)	3 (50.0)	1 (16.67)	0 (0)	0 (0)	0 (0)	6
Instrumental variable	5 (22.73)	2 (9.09)	3 (13.64)	2 (9.1)	4 (18.18)	1 (4.55)	3 (13.64)	2 (9.09)	22
Difference-in-differences (DiD)	33 (35.48)	24 (25.81)	8 (8.6)	7 (7.53)	11 (11.83)	4 (4.3)	2 (2.15)	4 (4.30)	93
Interrupted time series	17 (42.50)	6 (15.0)	5 (12.5)	6 (15.0)	1 (2.5)	3 (7.5)	2 (5.0)	0 (0)	40
Other	57 (55.34)	22 (21.4)	12 (11.65)	3 (2.91)	3 (2.91)	3 (2.91)	0 (0)	3 (2.91)	103
After comparison	12 (66.67)	2 (11.11)	0 (0)	1 (5.56)	1 (5.56)	1 (5.56)	1 (5.56)	0 (0)	18
Before-after comparison	59 (70.24)	5 (5.95)	6 (7.14)	7 (8.33)	4 (4.76)	1 (1.19)	1 (1.19)	1 (1.19)	84

### 3.3. Weaknesses and limitations of included studies

The primary weakness that was evident among the included studies was that none considered whether the *as-if* randomization assumption was satisfied and it was also challenging for readers to assess from the provided information whether this assumption was met. An additional weakness that we identified was the limited use of sensitivity analysis and falsification tests, which are important for strengthening causal inference.

## 4. Discussion

In this review we aimed to explore the practices and methods used in place-based NEE evaluations. This review of specific place-based NEEs of public health interventions has highlighted that their use as an evaluation tool in public health has accelerated since 2009. This trend echoes a recent similar observation in relation to social policies (30). We examined trends and developments in the use of methods and concepts for place-based NEEs, and their strength for causal inference. It was evident that there is lack of consistency in how a natural experiment was defined, and that studies sometimes self-identify as a natural experiment based on the characteristic that the intervention or the allocation of exposure was out of the researchers' control, regardless of the analysis approach used. However, a number of studies evaluated variation in exposure (similar to an observational study), rather than a specific change in exposure as a result of the occurrence/implementation of an intervention (akin to an RCT). This approach potentially weakens the potential for robust causal inference in many of the place-based NEE studies that we identified. We additionally observed that no studies considered whether the *as-if* randomization assumption was satisfied, while this was also difficult to assess from the provided information. Combined with the relatively limited use of sensitivity analyses and falsification tests, this has important implications for the extent to which such evaluations can be deemed to support causal inferences, and suggests an important way in which the reporting of NEEs could be improved.

Despite nearly 70% of the included studies evaluating a local or national policy, they employed a wide array of different study designs and analytic methods. 71% of studies used one of the well-defined natural experiment designs such as DiD studies, interrupted time series studies, before-after comparison studies, after comparison studies, instrumental variable studies, or synthetic control studies, but 29% used some other method; most often regression-based studies that explored variation in exposure and the change in exposure in the intervention group explicitly.

The exposure allocation process is critical to the design of natural experiment studies. This should mimic a random process as closely as possible to avoid biased inferences (14). However, authors rarely reported the plausibility of the *as-if* randomization assumption of exposure (12), leaving it to readers to assess this implicitly. Given that the *as-if* randomization assumption cannot be formally tested (16), we relied on reporting by the authors and by assessment of the reviewer based on the information provided. The research team classified only a minority (~ 2 in 5) of studies as having had plausible or likely *as-if* randomization, while one in four NEEs was rated as having implausible *as-if* randomization. These would not have been classified as natural experiments based on the definition by Dunning (12) [but might have based on the MRC definition (16)]. Assessing the plausibility of *as-if* randomization of exposure based on the information provided in the studies proved to be difficult, and the results of our small validation study of 18 NEEs, each assessed by 10 raters, had a poor inter-rater agreement. This indicates that *as-if*-random assessment is extremely difficult and unreliable, even for reviewers with particular experience in this topic. For comparison, a similar study was previously conducted to assess inter-rater agreement for risk of bias assessment using the Cochrane Collaboration Risk of bias tool. This study reported a higher ICC of 0.58 (95% CI 0.20–0.81) (31), compared to 0.35 (0.19–0.47) for *as-if* randomization in the current study. It remains unexplored whether this poor agreement results from the insufficient information provided in the publications, the raters themselves, or whether the process of judging the plausibility of *as-if* randomization itself is highly susceptible to error and bias. Given this difficulty in assessing *as-if* randomization, we suggest that the reporting of NEEs should include an assessment of the plausibly of this assumption by the authors, or should provide sufficient detail on the process of exposure allocation to enable readers to assess the likelihood of *as-if* randomization with a reasonable degree of confidence. Additionally, given that subjective judgments in these assessments will to some degree always be inevitable, causal inference from NEEs can be substantially increased by the inclusion of additional sensitivity analyses and falsification tests, ideally where these address different aspects of possible bias (14). This requires researchers to conduct such additional analyses, as the results of this review indicate only about half of the included studies reported any form of sensitivity or falsification analysis (10, 19).

Finally, this scoping review has further highlighted that there is limited standardization in the use and reporting of natural experiments. The evaluation of non-randomized interventions in public health would benefit from a more uniform approach for designing and appraising these evaluations, this approach could include direction on when certain approaches are more appropriate and when an evaluation could be classified as a natural experiment. This includes the use of the “Target Trial Framework”, which has been adapted for NEEs to support the design, reporting, and appraisal of NEEs (14). Furthermore, journals and reviewers could ensure publications adhere to these recommendations to enhance the quality of evidence identified as NEEs.

This extensive scoping review is the first to review the use of natural experiment evaluations of place-based interventions in public health, and as such the first review of its kind to demonstrate the range and trajectory of NEE use to support place-based evaluations in public health research. With over 350 studies included, this scoping review provides a comprehensive overview and insight into how place-based NEEs have been conducted and reported over the last two decades. Our review was conducted by a multidisciplinary review team with expertise in NEEs; systematic reviews, and intervention evaluation. We based our search strategy on a previously used strategy that looked to assess the use of systems science in evaluations of place-based public health interventions. In addition, we recognized the difficulty of assessing the *as-if* randomization of the assignment of exposure, and included the first validation exercise for the assessment of the “*as-if*-random” assumption.

This scoping review also has a number of limitations. We applied a cut-off date for our review of January 2020, and thus our data does not include additional NEEs published since. We reran the same search on 1 July 2021, which identified a further 2,167 studies for potential inclusion. Unfortunately, because of constraints on resources we were not able to include these additional studies. However, we note that the majority of these studies concerned evaluations of the many aspects of the Covid-19 pandemic, which we consider a unique and distinct topic area which is not comparable to the routine place-based public health interventions and policy evaluations identified here and would require its own review.

In addition to the use of the January 2020 cut-off date for inclusion, the review was limited to studies published in the English language. We recognized that the vast majority of studies will have been published in the English language and this review included NEEs from 54 countries, but nonetheless this is likely to have resulted in evidence from non-English speaking countries being underrepresented, including that from low and middle income countries. Additionally, search 3 included the most common study methods associated with natural experiments, however, we did not include all such methods. Although, search 3 was intended to pick up studies not already found in search 1 and 2, and we did not anticipate many to be found we acknowledge that by including only the most common study designs in search 3 we may have missed a few studies. Finally, for the inter-rater reliability exploration for the *as-if* randomization rating, it worth noting that although all authors that completed the rating have a common interest in the use of natural experiments for the evaluation of public health interventions, all come with a variety of backgrounds and expertise within public health. This may be a possible explanation for the low inter-rater reliability. Nonetheless, our scoping review provides a foundation to explore the use of natural experiment methodology in this field and more broadly in public health. Because of the large number of papers that were included we had to make the decision to have each NEE extracted by a single author (although PA double-checked a proportion of the coding in response to queries from others). This may have resulted in variations in coding.

Finally, we conducted an additional inter-rater assessment of *as-if* randomization of exposure for 18 randomly selected NEEs by 10 of the authors of this review. This showed poor agreement and demonstrated the difficulty of assessing this assumption from the information provided in the publications. In the absence of the “correct answers” or at least a “gold standard”, and despite all 10 authors having experience with conducting and appraising NEEs, we cannot exclude the possibility that the poor inter-rater agreement might have resulted from variability in the experience of the raters, or errors made in the process.

In addition to exploring the practices and methods used, trends and developments in the use of methods and concepts, and their strength for causal inference, we also aimed to provide recommendations for addressing the main weaknesses in published place-based NEEs in public health. Our review provides a strong motivation for consistent design and reporting of NEEs to facilitate the appraisal of the strengths and weaknesses of NEEs and thereby their potential to make causal claims. There are already a number of reporting guidelines that are applicable to NEEs or relevant specific study designs, or that could be adapted specifically for NEEs, for example STROBE, TREND (32, 33), TIDieR-PHP (34) and others (35, 36), and for design and reporting following the Target Trial Framework has also been proposed (14). Akin to RCTs, pre-registration of NEEs would further enhance methodological rigor. There are different schools of thoughts on the importance of *as-if* randomization for the definition of NEEs, but it is unquestionable that this is an important criteria for assessing the strength of any causal claims based upon such studies.

Although not directly addressed in the scoping review, NEEs can make important contributions to addressing health inequalities. For example, they can be important in investigating the determinants of health inequalities and in the identification of effective interventions to reduce inequalities (10); because they provide opportunities to study differential, subgroup effects. Especially for the evaluation of place-based public health interventions this offers particular benefits as geographical location data are often already available from routine data.

## 5. Conclusion

In conclusion, the scoping review demonstrates that NEEs are conducted using many different study designs and statistical methods and encompass various definitions of a natural experiment. It is questionable whether all evaluations reported as natural experiments should be considered as such, suggesting that the field would benefit from more consistent design and reporting of NEEs. Given the difficulty in assessing the likelihood of *as-if* randomization of the exposure, we recommend that researchers address this specifically in reports of NEEs by clearly demonstrating how social or political drivers behind the group assignment are indeed random. Further, we recommend that authors make more use of sensitivity/falsification tests or other robustness checks in the design and conduct of their studies, in order to improve their causal inference. We also recommend that reviewers should look for these design elements in the evaluation of funding applications and that research funders and journals should provide specific guidelines for what is expected in the design and reporting of NEEs.

## Data availability statement

The original contributions presented in the study are included in the article/Supplementary material, further inquiries can be directed to the corresponding author.

## Author contributions

FV conceived of the study. PA conducted the literature searches. PA and FV wrote the first version of the manuscript. All authors were involved in data extraction and analysis of the results, provided input in various iterations of the manuscript, and approved the final version.
