# Supplementary material for: Natural experiments for the evaluation of place-based public health interventions: a methodology scoping review
Source: Front Public Health. 2023 Jun 22;11:1192055. doi: 10.3389/fpubh.2023.1192055 (PMC10323422; doi:10.3389/fpubh.2023.1192055)
Supplement: Supplementary file 1 [file Data_Sheet_1.pdf]

Natural experiments for the evaluation of place-based public health interventions: a methodology scoping review

Patricia N Albers[1], Chiara Rinaldi[2], Heather Brown[3], Kate E Mason[4,5], Katrina d'Apice[1], Elizabeth McGill[2], Cheryl McQuire[1,6], Peter Craig[7], Anthony A Laverty[8], Morgan Beeson[9], Mhairi Campbell[7], Matt Egan[2], Marcia Gibson[7], Maxwell Fuller[1], Amy Dillon[1], David Taylor-Robinson[10], Russell Jago[1,11,12,], Kate Tilling [1,13], Benjamin Barr[14], Falko F Sniehotta[15,16], Matthew Hickman[1], Christopher J Millett[8], Frank de Vocht[1,11]

[1]Population Health Sciences, Bristol Medical School, the University of Bristol. Bristol, United Kingdom
[2]Department of Public Health, Environments and Society, London School of Hygiene and Tropical Medicine, United Kingdom
[3]Health Research, Lancaster University, United Kingdom
[4]Department of Public Health Policy & Systems, University of Liverpool
[5]Centre for Health Policy, University of Melbourne
[6]National Institute for Health and Care Research (NIHR) School for Public Health Research (SPHR)
[7]MRC/CSO Social and Public Health Sciences Unit, School of Health and Wellbeing, University of Glasgow.
[8]School of Public Health, Imperial College London
[9]Newcastle University Business School, Newcastle University
[10]Department of Public Health, Policy and Systems. University of Liverpool
[11]NIHR Applied Research Collaboration West (NIHR ARC West)
[12]Centre for Exercise, Nutrition & Health Sciences, School for Policy Studies, University of Bristol
[13]MRC Integrative Epidemiology Unit, University of Bristol
[14]Institute of Population Health, University of Liverpool
[15]NIHR Policy Research Unit Behavioural Science, Newcastle University
[16]Department of Public Health, Social and Preventive Medicine, Medical Faculty Mannheim, Heidelberg University.

Supplementary Materials

Table S1. Literature search strategies

Search:	Terms
1	1) Natural experiments:<br>Natural experiment*<br>AND<br>2) Evaluation:<br>Evalat* OR “policy evaluat*” OR “prog* evaluat*” OR “formative evaluat*” OR “process evaluat*” OR “outcome evaluat” OR context evaluat* OR evaluation studies as topic/ OR programme evaluation/<br>AND<br>3) public health (*)<br>("public health" OR "health promotion" OR "health inequality*" OR "health inequalities*" OR “health inequity” OR “health inequalities” OR "health behavio?r" OR "well-being" OR wellbeing OR nutrition OR obesity OR "fast food*" OR sugar OR salt OR tobacco OR smoking OR cigarette* OR alcohol OR "illegal drug*" OR "illicit drug*" OR "recreational drug*" OR "social determinant*" OR crime OR “community safety” OR transport* OR “urban health”)
2	1) Natural experiments:<br>“Natural experiment*”<br>AND<br>3) public health (*)<br>("public health" OR "health promotion" OR "health inequality*" OR “health improvement” OR “health protection” OR "health inequalities*" OR “health inequity” OR “health inequalities” OR "health behavio?r" OR "well-being" OR wellbeing OR nutrition OR obesity OR "fast food*" OR sugar OR salt OR tobacco OR smoking OR cigarette* OR alcohol OR "illegal drug*" OR "illicit drug*" OR "recreational drug*" OR "social determinant*" OR crime OR “community safety” OR transport* OR “urban health”)
3	1) Natural experiments:

	“interrupted time series” OR “controlled interrupted time series” OR “ITS” OR “controlled ITS” OR “before-after” OR “synthetic control*” OR “instrumental variable*”<br>AND<br>3) public health (*)<br>("public health" OR "health promotion" OR "health inequality*" OR “health improvement” OR “health protection” OR "health inequalities*" OR “health inequity” OR “health inequalities” OR "health behavio?r" OR "well-being" OR wellbeing OR nutrition OR obesity OR "fast food*" OR sugar OR salt OR tobacco OR smoking OR cigarette* OR alcohol OR "illegal drug*" OR "illicit drug*" OR "recreational drug*" OR "social determinant*" OR crime OR “community safety” OR transport* OR “urban health”)

Table S2. List of all place-based natural experiment evaluations included in scoping review

Reference number	Publication Title	Lead Authors	Publication Year
1	Effects of Intimate Partner Physical Violence on Newborns' Birth Outcomes Among Jordanian Birthing Women	Abujilban, S. et al.	2017
2	1918 Influenza Pandemic: In Utero Exposure in the United States and Long-Term Impact on Hospitalizations	Acquah, J. K. et al.	2017
3	Associations between introduction and withdrawal of a financial incentive and timing of attendance for antenatal care and incidence of small for gestational age: natural experimental evaluation using interrupted time series methods	Adams, J. et al.	2018
4	Adopting Clean Fuels and Technologies on School Buses. Pollution and Health Impacts in Children	Adar, S. D. et al.	2015
5	User charges and the demand for acute paediatric traumatology services	Ahlamaa-Tuompo, J. et al.	1998
6	The impact of flooding on marriage: evidence from Pakistan	Ahmed, R.	2018
7	Socio-Ecological Natural Experiment with Randomized Controlled Trial to Promote Active Commuting to Work: Process Evaluation, Behavioral Impacts, and Changes in the Use and Quality of Walking and Cycling Paths	Aittasalo, M. et al.	2019
8	Adult cardiopulmonary mortality and indoor air pollution: a 10-year retrospective cohort study in a low-income rural setting	Alam, D. S. et al.	2012
9	Impact of the phased abolition of co-payments on the utilisation of selected prescription medicines in Wales	Alam, M. F. et al.	2018
10	Changes in mental health status amongst children of migrants to Australia: a longitudinal study	Alati, R. et al.	2003
11	Chernobyl's Subclinical Legacy: Prenatal Exposure to Radioactive Fallout and School Outcomes in Sweden	Almond, D. et al.	2009
12	In Utero Ramadan Exposure and Children's Academic Performance	Almond, D. et al.	2014
13	Evaluating Saudi Arabia’s 50% carbonated drink excise tax: Changes in prices and volume sales	Alsukait, R. et al.	2020
14	Democratisation and health after the fall of the Wall	Alvarez-Dardet, C. et al.	2006

15	Increases in Use and Activity Due to Urban Renewal: Effect of a Natural Experiment	Andersen, H. B. et al.	2017
16	Drinking Alone? The Effect of an Alcohol Treatment Program on Relationship Stability for Convicted Drunk Drivers in Denmark	Andersen, S. H.	2016
17	Urban sprawl and body mass index among displaced Hurricane Katrina survivors	Arcaya, M. et al.	2014
18	A population based study comparing changes in rotavirus burden on the Island of Ireland between a highly vaccinated population and an unvaccinated population	Armstrong, G. et al.	2016
19	The non-take up of long-term care benefit in France: A pecuniary motive?	Arrighi, Y. et al.	2015
20	The Impact of Activity Based Working (ABW) on Workplace Activity, Eating Behaviours, Productivity, and Satisfaction	Arundell, L. et al.	2018
21	Impact of the 2008 Beijing Olympics on the risk of pregnancy complications	Assibey-Mensah, V. et al.	2016
22	The impact of cost-sharing schemes on drug compliance in Italy: evidence based on quantile regression	Atella, V. et al.	2014
23	The Effect of Changes in State and Federal Policy Fornonprescription Access to Emergency Contraception Onyouth Contraceptive Use: A Difference-in-Difference Analysis across New England States	Atkins, D. N. et al.	2014
24	The impact of expanded health insurance coverage for unauthorized pregnant women on prenatal care utilization	Atkins, D. N. et al.	2018
25	Changes in physical activity after building a greenway in a disadvantaged urban community: A natural experiment	Auchincloss, A. H. et al.	2019
26	Do flexible work policies improve parents' health? A natural experiment based on the UK Millennium Cohort Study	Avendano, M. et al.	2018
27	County Smoke-Free Laws and Cigarette Smoking Among U.S. Adults, 1995-2015	Azagba, S. et al.	2020
28	The effect of dance mat exergaming systems on physical activity and health-related outcomes in secondary schools: results from a natural experiment	Azevedo, L. B. et al.	2014
29	Australian Firearm Related Deaths: New Findings and Implications for Crime Prevention and Health Policies following revisions to Official Death Count Data	Baker, J. et al.	2015
30	Trade and investment liberalization, food systems change and highly processed food consumption: a natural experiment contrasting the soft-drink markets of Peru and Bolivia	Baker, P. et al.	2016
31	The effect of education on health: Evidence from the 1997 compulsory schooling reform in Turkey	Baltagi, B. H. et al.	2019
32	Education can reduce health differences related to genetic risk of obesity	Barcellos, S. H. et al.	2018

33	The Impact of U.S. Free Trade Agreements on Calorie Availability and Obesity: A Natural Experiment in Canada	Barlow, P. et al.	2018
34	Implementation of a Lifestyle Intervention for People With Serious Mental Illness in State-Funded Mental Health Centers	Bartels, S. J. et al.	2018
35	Medical Marijuana Laws and Suicide	Bartos, B. J. et al.	2019
36	Prostate cancer mortality after introduction of prostate-specific antigen mass screening in the Federal State of Tyrol, Austria	Bartsch, G. et al.	2001
37	Taking up cycling after residential relocation: built environment factors	Beenackers, M. A. et al.	2012
38	The effect of increased primary schooling on adult women's HIV status in Malawi and Uganda: Universal Primary Education as a natural experiment	Behrman, J. A.	2015
39	Does Keeping Adolescent Girls in School Protect Against Sexual Violence? Quasi-Experimental Evidence From East and Southern Africa	Behrman, J. A. et al.	2017
40	Association of implementation of a public bicycle share program with intention and self-efficacy: The moderating role of socioeconomic status	Belanger-Gravel, A. et al.	2016
41	Evaluation of a complex healthcare intervention to increase smoking cessation in pregnant women: interrupted time series analysis with economic evaluation	Bell, R. et al.	2018
42	A community-based intervention increases physical activity and reduces obesity in school-age children in North Carolina	Neelon, B. et al.	2015
43	Alcohol control and injury death in Alaska native communities: wet, damp and dry under Alaska's local option law	Berman, M. et al.	2000
44	Do Consumers Exploit Commitment Opportunities? Evidence from Natural Experiments Involving Liquor Consumption	Bernheim, B. D. et al.	2016
45	The enduring influence of drinking motives on alcohol consumption after fateful trauma	Beseler, C. L. et al.	2011
46	Early-Life Exposure to the Great Smog of 1952 and the Development of Asthma	Bharadwaj, P. et al.	2016
47	Changes in Alcohol-Related Harm after Alcohol Policy Changes in Denmark	Bloomfield, K. et al.	2009
48	Liquor-by-the-drink and alcohol-related traffic crashes: a natural experiment using time-series analysis	Blose, J. O. et al.	1987
49	Does playground improvement increase physical activity among children? A quasi-experimental study of a natural experiment	Bohn-Goldbaum, E. E. et al.	2013
50	Cattle eradication and malnutrition in under five's: a natural experiment in Botswana	Boonstra, E. et al.	2001
51	The effectiveness of a 20 mph speed limit intervention on vehicle speeds in Bristol, UK: A non-randomised stepped wedge design	Bornioli, A. et al.	2018
52	Effects of city-wide 20 mph (30km/hour) speed limits on road injuries in Bristol, UK	Bornioli, A. et al.	2019

53	External validation of the Cardiff model of information sharing to reduce community violence: natural experiment	Boyle, A. A. et al.	2013
54	The causal relationship between education, health and health related behaviour: evidence from a natural experiment in England	Braakmann, N.	2011
55	Cannabis depenalisation, drug consumption and crime - evidence from the 2004 cannabis declassification in the UK	Braakmann, N. et al.	2014
56	Hospital Admissions for Myocardial Infarction and Stroke Before and After the Trans-Fatty Acid Restrictions in New York	Brandt, E. J. et al.	2017
57	Spatial spillover effects of a community action programme targeting on-licensed premises on violent assaults: evidence from a natural experiment	Brannstrom, L. et al.	2016
58	Causes and consequences of teen childbearing: Evidence from a reproductive health intervention in South Africa	Branson, N. et al.	2018
59	State School Policies as Predictors of Physical and Mental Health: A Natural Experiment in the REGARDS Cohort	Brenowitz, W. D. et al.	2019
60	Cigarette brand diversity and price changes during the implementation of plain packaging in the United Kingdom	Breton, M. O. et al.	2018
61	Impacts of active school design on school-time sedentary behavior and physical activity: A pilot natural experiment	Brittin, J. et al.	2017
62	Before and After a New Light Rail Stop: Resident Attitudes, Travel Behavior, and Obesity	Brown, B. B. et al.	2009
63	Positive income shocks and accidental deaths among Cherokee Indians: a natural experiment	Bruckner, T. A. et al.	2011
64	The psychological cost of aircraft noise for children	Bullinger, M. et al.	1999
65	Thank you for not smoking: Evidence from the Italian smoking ban	Buonanno, P. et al.	2012
66	The Oakville Oil Refinery Closure and Its Influence on Local Hospitalizations: A Natural Experiment on Sulfur Dioxide	Burr, W. S. et al.	2018
67	The effect of a neighborhood built environment on physical activity behaviors	Calise, T. V. et al.	2012
68	Release from drinking-age restrictions is associated with increases in alcohol-related motor vehicle collisions among young drivers in Canada	Callaghan, R. C. et al.	2016
69	Do health checks for adults with intellectual disability reduce emergency hospital admissions? Evaluation of a natural experiment	Carey, I. M. et al.	2017
70	Income inequality in uptake of voluntary versus organised breast cancer screening: evidence from the British Household Panel Survey	Carney, P. et al.	2018
71	Increase in fertility following coal and oil power plant retirements in California	Casey, J. A. et al.	2018

72	Retirements of Coal and Oil Power Plants in California: Association With Reduced Preterm Birth Among Populations Nearby	Casey, J. A. et al.	2018
73	Changes in prevalence of prescription opioid abuse after introduction of an abuse-deterrent opioid formulation	Cassidy, T. A. et al.	2014
74	The impact of income on the weight of elderly Americans	Cawley, J. et al.	2010
75	Reducing violence by transforming neighborhoods: a natural experiment in Medellin, Colombia	Cerda, M. et al.	2012
76	When War Comes Home: The Effect of Combat Service on Domestic Violence	Cesur, R. et al.	2016
77	Effectiveness of Food Subsidies in Raising Healthy Food Consumption: Public Distribution of Pulses in India	Chakrabarti, S. et al.	2018
78	The role of formal schooling on weight in young children	Chang, C. Y. et al.	2017
79	Sharing meals with institutionalized people with dementia: a natural experiment	Charras, K. et al.	2010
80	A longitudinal examination of a pay-for-performance program for diabetes care: evidence from a natural experiment	Cheng, S-H. et al.	2012
81	National health insurance and life satisfaction in late life: longitudinal findings from a natural experiment in Taiwan	Chiao, C. et al.	2014
82	Parental Education and Child Health: Evidence from a Natural Experiment in Taiwan	Chou, S. Y. et al.	2010
83	A new urban planning code's impact on walking: the residential environments project	Christian, H. et al.	2013
84	A Longitudinal Analysis of the Influence of the Neighborhood Environment on Recreational Walking within the Neighborhood: Results from RESIDE	Christian, H. et al.	2017
85	Indoor tobacco legislation is associated with fewer emergency department visits for asthma exacerbation in children	Ciaccio, C. E. et al.	2016
86	Variation across Romania in the health impact of increasing tobacco taxation	Ciobanu, M. et al.	2018
87	Deterrence and fare evasion: Results of a natural experiment	Clarke, R. V. et al.	2010
88	Alcohol outlets, gonorrhea, and the Los Angeles civil unrest: a longitudinal analysis	Cohen, D. A. et al.	2006
89	New Recreational Facilities for the Young and the Old in Los Angeles: Policy and Programming Implications	Cohen, D. A. et al.	2009
90	Current affairs and the public psyche: American anxiety in the post 9/11 world	Cohen, P. et al.	2006
91	Is breast truly best? Estimating the effects of breastfeeding on long-term child health and wellbeing in the United States using sibling comparisons	Colen, C. G. et al.	2014
92	Translating evidence-based falls prevention into clinical practice in nursing facilities: Results and lessons from a quality improvement collaborative	Colon-Emeric, C. et al.	2006

93	Change in non-alcoholic beverage sales following a 10-pence levy on sugar-sweetened beverages within a national chain of restaurants in the UK: interrupted time series analysis of a natural experiment	Cornelsen, L. et al.	2017
94	Association of family income supplements in adolescence with development of psychiatric and substance use disorders in adulthood among an American Indian population	Costello, E. J. et al.	2010
95	Who Benefits from Repealing Tampon Taxes? Empirical Evidence from New Jersey	Cotropia, C. et al.	2018
96	Longer schooling but not better off? A quasi-experimental study of the effect of compulsory schooling on biomarkers in France	Courtin, E. et al.	2019
97	Long-term effects of compulsory schooling on physical, mental and cognitive ageing: a natural experiment	Courtin, E. et al.	2019
98	Impact of an outdoor gym on park users' physical activity: A natural experiment	Cranney, L. et al.	2016
99	Boosting workplace wellness programs with financial incentives	Cuellar, A. et al.	2017
100	Large scale food retailing as an intervention for diet and health: quasi-experimental evaluation of a natural experiment	Cummins, S. et al.	2005
101	Use and cost effectiveness of smoking-cessation services under four insurance plans in a health maintenance organization	Curry, S. J. et al.	1998
102	Natural experiment examining the longitudinal association between change in residential segregation and youth cardiovascular health across race/ethnicity and gender in the USA	D'Agostino, E. M. et al.	2018
103	Does transportation vulnerability explain the relationship between changes in exposure to segregation and youth cardiovascular health?	D'Agostino, E. M. et al.	2019
104	Regional Cooperative Pollution Control and Residents' Health Expenditures: Empirical Evidence from China	Dai, Y. et al.	2019
105	The Effect of State Competitive Food and Beverage Regulations on Childhood Overweight and Obesity	Datar, A. et al.	2017
106	Length of secondary schooling and risk of HIV infection in Botswana: evidence from a natural experiment	De Neve, J-W. et al.	2015
107	Causal Effect of Parental Schooling on Early Childhood Undernutrition: Quasi-Experimental Evidence From Zimbabwe	De Neve, J-W. et al.	2018
108	Evaluating the causal impact of individual alcohol licensing decisions on local health and crime using natural experiments with synthetic controls	de Vocht, F. et al.	2020
109	The intervention effect of local alcohol licensing policies on hospital admission and crime: a natural experiment using a novel Bayesian synthetictime-series method	de Vocht, F. et al.	2017

110	Bicycle boulevards and changes in physical activity and active transportation: findings from a natural experiment	Dill, J. et al.	2014
111	Causal Effect of Sleep Duration on Body Weight in Adolescents: A Population-based Study Using a Natural Experiment	Do, Y. K.	2019
112	Taking the pressure off the spring: the case of rebounding smoking rates when antitobacco campaigns ceased	Dono, J. et al.	2019
113	The relationship between trees and human health: evidence from the spread of the emerald ash borer	Donovan, G. H. et al.	2013
114	Health insurance and child mortality in Costa Rica	Dow, W. H. et al.	2003
115	Abolishing Fees at Health Centers in the Context of Community Case Management of Malaria: What Effects on Treatment-Seeking Practices for Febrile Children in Rural Burkina Faso?	Druetz, T. et al.	2015
116	Results from a natural experiment: initial neighbourhood investments do not change objectively-assessed physical activity, psychological distress or perceptions of the neighbourhood	Dubowitz, T. et al.	2019
117	Assessment of Exposure to High-Performing Schools and Risk of Adolescent Substance Use: A Natural Experiment	Dudovitz, R. N. et al.	2018
118	Access to alcohol and heart disease among patients in hospital: observational cohort study using differences in alcohol sales laws	Dukes, J. W. et al.	2016
119	The Impact of Education on Health Outcomes and Behaviors in a Middle-Income, Low-Education Country	Dursun, B. et al.	2018
120	Health impacts of free bus travel for young people: evaluation of a natural experiment in London	Edwards, P. et al.	2013
121	Health effects of neighborhood demolition and housing improvement: a prospective controlled study of 2 natural experiments in urban renewal	Egan, M. et al.	2013
122	Proportionate universalism in practice? A quasi-experimental study (GoWell) of a UK neighbourhood renewal programme's impact on health inequalities	Egan, M. et al.	2016
123	Environmental change and infectious disease: how new roads affect the transmission of diarrheal pathogens in rural Ecuador	Eisenberg, J. N. S. et al.	2006
124	Supermarket policies on less-healthy food at checkouts: Natural experimental evaluation using interrupted time series analyses of purchases	Ejlerskov, K. T. et al.	2018
125	Child and adolescent fast-food choice and the influence of calorie labeling: a natural experiment	Elbel, B. et al.	2011
126	The Good Food Junction: a Community-Based Food Store Intervention to Address Nutritional Health Inequities	Engler-Stringer, R. et al.	2016

127	Enrolment of families with overweight children into a program aimed at reducing childhood obesity with and without a weight criterion: a natural experiment	Esdaile, E. et al.	2019
128	The benefits of prenatal care: evidence from the PAT bus strike	Evans, W. N. et al.	2005
129	General deterrence of drunk driving: evaluation of recent American policies	Evans, W. N. et al.	1991
130	The impact of civil union legislation on minority stress, depression, and hazardous drinking in a diverse sample of sexual-minority women: A quasi-natural experiment	Everett, B. G. et al.	2016
131	Are tobacco control policies effective in reducing young adult smoking?	Farrelly, M. C. et al.	2014
132	Food and beverage price discounts to improve health in remote Aboriginal communities: mixed method evaluation of a natural experiment	Ferguson, M. et al.	2017
133	Impact of a major disaster on the mental health of a well-studied cohort	Fergusson, D. M. et al.	2014
134	How do individuals' health behaviours respond to an increase in the supply of health care? Evidence from a natural experiment	Fichera, E. et al.	2016
135	Wartime Paris, cirrhosis mortality, and the Ceteris paribus assumption	Fillmore, K. M. et al.	2002
136	Identifying the effect of shelf nutrition labels on consumer purchases: results of a natural experiment and consumer survey	Finkelstein, E. A. et al.	2018
137	Urban trails and physical activity: a natural experiment	Fitzhugh, E. C. et al.	2010
138	A natural experiment on the impact of fruit supplementation on asthma symptoms in children	Fogarty, A. W. et al.	2009
139	Longitudinal association between change in the neighbourhood built environment and the wellbeing of local residents in deprived areas: an observational study	Foley, L. et al.	2018
140	Effects of living near an urban motorway on the wellbeing of local residents in deprived areas: Natural experimental study	Foley, L. et al.	2017
141	Effects of living near a new urban motorway on the travel behaviour of local residents in deprived areas: Evidence from a natural experimental study	Foley, L. et al.	2017
142	The Role of School Design in Shaping Healthy Eating-Related Attitudes, Practices, and Behaviors Among School Staff	Frerichs, L et al.	2016
143	Impact of a public transit strike on public bicycle share use: An interrupted time series natural experiment study	Fuller, D. et al.	2019
144	Effects of land titling on child health	Galiani, S. et al.	2004
145	Do Neighborhood Effects on Low-Income Minority Children Depend on Their Age? Evidence from a Public Housing Natural Experiment	Galster, G. et al.	2017
146	Effects of tobacco control policy on cardiovascular morbidity and mortality in Russia	Gambaryan, M. et al.	2018

147	Comparative effectiveness of after-school programs to increase physical activity	Gesell, S. B. et al.	2013
148	The impact of Universal Health Coverage on health care consumption and risky behaviours: evidence from Thailand	Ghislandi, S. et al.	2015
149	What happens to diet and child health when migration splits households? Evidence from a migration lottery program	Gibson, J. et al.	2011
150	Natural experiment evidence on the effect of migration on blood pressure and hypertension	Gibson, J. et al.	2013
151	The influence of urban design on neighbourhood walking following residential relocation: longitudinal results from the RESIDE study	Giles-Corti, B. et al.	2013
152	Does childhood schooling affect old age memory or mental status? Using state schooling laws as natural experiments	Glymour, M. M. et al.	2008
153	Evaluation of Nischay scheme in improving antenatal care in a northern state of India	Goel, S. et al.	2013
154	Daylight saving time as a potential public health intervention: an observational study of evening daylight and objectively-measured physical activity among 23,000 children from 9 countries	Goodman, A. et al.	2014
155	Effectiveness and equity impacts of town-wide cycling initiatives in England: a longitudinal, controlled natural experimental study	Goodman, A. et al.	2013
156	Impact of offering cycle training in schools upon cycling behaviour: a natural experimental study	Goodman, A. et al.	2016
157	Impact of Austria's 2009 trans fatty acids regulation on all-cause, cardiovascular and coronary heart disease mortality	Grabovac, I. et al.	2018
158	Do Medicaid physician fees for prenatal services affect birth outcomes?	Gray, B.	2001
159	Patterns of pre-crash behaviour in railway suicides and the effect of corridor fencing: a natural experiment in New South Wales	Gregor, S. et al.	2019
160	Fetal malnutrition and academic success: Evidence from Muslim immigrants in Denmark	Greve, J. et al.	2017
161	Closing the loop: short term impacts on physical activity of the completion of a loop trail in Sydney, Australia	Grunseit, A. et al.	2019
162	Do Patients With More Education Receive More Subsidized Dental Care? Evidence From a Natural Experiment Using the Introduction of a School Reform in Norway as an Instrumental Variable	Grytten, J. et al.	2018
163	The Impact of Female Education on Teenage Fertility: Evidence from Turkey	Gunes, P. M.	2015
164	Impacts on Emergency Department Visits from Personal Responsibility Provisions: Evidence from West Virginia's Medicaid Redesign	Gurley-Calvez, T. et al.	2016

165	The effect of ingredient-specific calorie information on calories ordered	Gustafson, C. R. et al.	2018
166	Changes in Alcohol Availability, Price and Alcohol-related Problems and the Collectivity of Drinking Cultures: What Happened in Southern and Northern Sweden?	Gustafsson, N. K.	2010
167	Universal Child Care and Long-Term Effects on Child Well-Being: Evidence from Canada	Haeck, C. et al.	2018
168	Paid Parental Leave: Leaner Might Be Better	Haeck, C. et al.	2019
169	An evaluation of the effects of lowering blood alcohol concentration limits for drivers on the rates of road traffic accidents and alcohol consumption: a natural experiment	Haghpanahan, H. et al.	2019
170	Changing national guidelines is not enough: the impact of 1990 IOM recommendations on gestational weight gain among US women	Hamad, R. et al.	2016
171	Educational attainment and cardiovascular disease in the United States: A quasi-experimental instrumental variables analysis	Hamad, R, et al.	2019
172	Does Free Public Health Care Increase Utilization and Reduce Spending? Heterogeneity and Long Term Effects	Hangoma, P, et al.	2018
173	Do state breastfeeding laws in the USA promote breast feeding?	Hawkins, S. S. et al.	2013
174	Changes in alcohol consumption following a reduction in the price of spirits: a natural experiment in Switzerland	Heeb, J-L. et al.	2003
175	Evaluation of the Veloway 1: A natural experiment of new bicycle infrastructure in Brisbane, Australia	Heesch, K. C. et al.	2016
176	Changes in alcohol-related mortality and its socioeconomic differences after a large reduction in alcohol prices: a natural experiment based on register data	Herttua, K. et al.	2008
177	The effects of a large reduction in alcohol prices on hospitalizations related to alcohol: a population-based natural experiment	Herttua, K. et al.	2011
178	An evaluation of the impact of a large reduction in alcohol prices on alcohol-related and all-cause mortality: time series analysis of a population-based natural experiment	Herttua, K. et al.	2011
179	Educational inequalities in hospitalization attributable to alcohol: a population-based longitudinal study of changes during the period 2000-07	Herttua, K. et al.	2015
180	Minimum Prices for Alcohol and Educational Disparities in Alcohol-related Mortality	Herttua, K. et al.	2014
181	The impact of a large reduction in the price of alcohol on area differences in interpersonal violence: a natural experiment based on aggregate data	Herttua, K. et al.	2008
182	Living alone and alcohol-related mortality: a population-based cohort study from Finland	Herttua, K. et al.	2011
183	Effect of expanded US recommendations for seasonal influenza vaccination: comparison of two pediatric emergency departments in the United States and Canada	Hoen, A. G. et al.	2011

184	Evaluating the impact of implementing public bicycle share programs on cycling: the International Bikeshare Impacts on Cycling and Collisions Study (IBICCS)	Hosford, K.et al.	2019
185	Ambient air pollution and adverse birth outcomes: a natural experiment study	Huang, C. et al.	2015
186	The Impacts of China's Urban Employee Basic Medical Insurance on Healthcare Expenditures and Health Outcomes	Huang, F. et al.	2017
187	Do flexible alcohol trading hours reduce violence? A theory-based natural experiment in alcohol policy	Humphreys, D. K. et al.	2014
188	Evaluating the impact of flexible alcohol trading hours on violence: an interrupted time series analysis	Humphreys, D. K. et al.	2013
189	Examining the impact of a summer learning program on children's weight status and cardiorespiratory fitness: A natural experiment	Hunt, E. T. et al.	2019
190	Thresholds of socio-economic and environmental conditions necessary to escape from childhood malnutrition: a natural experiment in rural Gambia	Husseini, M. et al.	2018
191	Incentives in a Medicaid carve-out: impact on children with special health care needs	Inkelas, M.	2005
192	Suicide prevention--a medical breakthrough?	Isacsson, G.	2000
193	Daylight saving time shifts and incidence of acute myocardial infarction--Swedish Register of Information and Knowledge About Swedish Heart Intensive Care Admissions (RIKS-HIA)	Janszky, I. et al.	2012
194	Medicaid enrollment policy increased smoking cessation among pregnant women but had no impact on birth outcomes	Jarlenski, M. et al.	2014
195	Effects of new dock-less bicycle-sharing programs on cycling: a retrospective study in Shanghai	Jia, Y. et al.	2019
196	Association Between Casino Opening or Expansion and Risk of Childhood Overweight and Obesity	Jones-Smith, J. C. et al.	2014
197	Association between Native American-owned casinos and the prevalence of large-for-gestational-age births	Jones-Smith, J C. et al.	2017
198	Cigarette ignition propensity, smoking behavior, and toxicant exposure: A natural experiment in Canada	June, K. M. et al.	2011
199	Environmental influences on young adult weight gain: evidence from a natural experiment	Kapinos, K. et al.	2011
200	Obesogenic environmental influences on young adults: evidence from college dormitory assignments	Kapinos, K. et al.	2014
201	Employees' drug purchases before and after organizational downsizing: a natural experiment on the Norwegian working population (2004-2012)	Kaspersen, S. L. et al.	2017

202	Effects of restrictions to Income Support on health of lone mothers in the UK: a natural experiment study	Katikireddi, S. V. et al.	2018
203	Do food stamps cause obesity? Evidence from immigrant experience	Kaushal, N.	2007
204	Changes in compulsory schooling and the causal effect of education on health: evidence from Germany	Kemptner, D. et al.	2011
205	Heat-Related Mortality in Japan after the 2011 Fukushima Disaster: An Analysis of Potential Influence of Reduced Electricity Consumption	Kim, Y. et al.	2017
206	Effect of a vitamin and mineral supplementation on glycemic status: Results from a community-based program	Kimball, S. M. et al.	2017
207	Six physical education lessons a week can reduce cardiovascular risk in school children aged 6-13 years: a longitudinal study	Klakk, H. et al.	2014
208	The effect of tobacco control policy on smoking cessation in relation to gender, age and education in Lithuania, 1994-2010	Klumbiene, J. et al.	2015
209	Changes in workplace car parking and commute mode: a natural experimental study	Knott, C. S. et al.	2019
210	Abortion legislation, maternal healthcare, fertility, female literacy, sanitation, violence against women and maternal deaths: a natural experiment in 32 Mexican states	Koch, E. et al.	2015
211	Impacts of Federally Funded State Obesity Programs on Adult Obesity Prevalence in the United States, 1998-2010	Koh, K. et al.	2018
212	The 18-month impact of special supplemental nutrition program for women, infants, and children food package revisions on diets of recipient families	Kong, A. et al.	2014
213	The shattered "Iron Rice Bowl": Intergenerational effects of Chinese State-Owned Enterprise reform	Kong, N. et al.	2019
214	Alcohol-related mortality in Ukraine	Krasovsky, K.	2009
215	The effect of early postpartum home visits by health visitors: a natural experiment	Kronborg, H. et al.	2012
216	Widening educational inequalities in adolescent smoking following national tobacco control policies in the Netherlands in 2003: a time-series analysis	Kuipers, M. A. G. et al.	2014
217	The effect of universal influenza immunization on mortality and health care use	Kwong, J. C. et al.	2008
218	Causal effect of education on mortality in a quasi-experiment on 1.2 million Swedes	Lager, A. C. J. et al.	2012
219	Restricting access to a suicide hotspot does not shift the problem to another location. An experiment of two river bridges in Brisbane, Australia	Law, C-K. et al.	2014
220	The effect of labor market shocks on health: The case of the Russian transition	Lazareva, O. et al.	2020
221	Early-Life Assets in Oldest-Old Age: Evidence From Primary Care Reform in Early Twentieth Century Sweden	Lazuka, V. et al.	2019

222	School Breakfast-Club Program Changes and Youth Eating Breakfast During the School Week in the COMPASS Study	Leatherdale, S. T. et al.	2016
223	Does more education lead to better health habits? Evidence from the school reforms in Australia	Li, J. H. et al.	2015
224	Mortality benefits of vigorous air quality improvement interventions during the periods of APEC Blue and Parade Blue in Beijing, China	Lin, H. et al.	2017
225	Affordable Care Act and cancer stage at diagnosis in an underserved population	Lu, Y. et al.	2019
226	Handgun waiting periods reduce gun deaths	Luca, M. et al.	2017
227	Valuing Air Quality Using the Life Satisfaction Approach	Luechinger, S.	2009
228	Gambling behaviour and the prevalence of gambling problems in adult EGM gamblers when EGMs are banned. A natural experiment	Lund, I.	2009
229	Health Care Access and Receipt of Clinical Diabetes Preventive Care for Working-Age Adults With Diabetes in States With and Without Medicaid Expansion: Results from the 2013 and 2015 BRFSS	Luo, H. et al.	2019
230	School-based body mass index screening and parent notification: a statewide natural experiment	Madsen, K. A.	2011
231	The incentives created by a harm reduction approach to smoking cessation: Snus and smoking in Sweden and Finland	Maki, J.	2015
232	Using surveillance data to inform community action: the effect of alcohol sale restrictions on intentional injury-related ambulance pickups	Masho, S. W. et al.	2014
233	Learning to Trust Flu Shots: Quasi-Experimental Evidence from the 2009 Swine Flu Pandemic	Maurer, J. et al.	2016
234	Evaluation of a health promoting schools program in a school board in Nova Scotia, Canada	McIsaac, J-L. D. et al.	2017
235	Exploring the short-term impact of community water fluoridation cessation on children's dental caries: a natural experiment in Alberta, Canada	McLaren, L. et al.	2017
236	More COPS, less crime	Mello, S.	2019
237	Cesarean delivery rates, costs and readmission of childbirth in the new cooperative medical scheme after implementation of an episode-based bundled payment (EBP) policy	Meng, Z. et al.	2019
238	An evaluation of school-based e-cigarette control policies' impact on the use of vaping products	Milicic, S. et al.	2018
239	New physical activity spaces in deprived neighborhoods: Does it change outdoor play and sedentary behavior? A natural experiment	Molenberg, F. J. M. et al.	2019

240	Effect of an annual benefit limit on adult dental expenditure and utilization: a cross-sectional analysis	Morrison, G. C. et al.	2012
241	SNAP Participation Improves Children's Health Care Use: An Analysis of ARRA's Natural Experiment	Morrissey, T. W. et al.	2020
242	Social equity in Human Papillomavirus vaccination: a natural experiment in Calgary Canada	Musto, R. et al.	2013
243	Sex-selective Abortion Bans are Not Associated with Changes in Sex Ratios at Birth among Asian Populations in Illinois and Pennsylvania	Nandi, A. et al.	2015
244	Greater drug injecting risk for HIV, HBV, and HCV infection in a city where syringe exchange and pharmacy syringe distribution are illegal	Neaigus, A. et al.	2008
245	Effect of innovative building design on physical activity	Nicoll, G. et al.	2009
246	Neighborhood Environments and Physical Activity: A Longitudinal Study of Adolescents in a Natural Experiment	Nicosia, N. et al.	2018
247	Assessing the impact of media guidelines for reporting on suicides in Austria: interrupted time series analysis	Niederkrotenthaler, T. et al.	2007
248	Decline in breast cancer mortality: how much is attributable to screening?	Njor, S. H. et al.	2015
249	Long-term effects of minimum drinking age laws on past-year alcohol and drug use disorders	Norberg, K. E. et al.	2009
250	Saturday opening of alcohol retail shops in Sweden: an experiment in two phases	Norstrom, T. et al.	2005
251	Evaluating the initial impact of the revised Special Supplemental Nutrition Program for Women, Infants, and Children (WIC) food packages on dietary intake and home food availability in African-American and Hispanic families	Odoms-Young, A. M. et al.	2014
252	Biological welfare and the commons: A natural experiment in the Alps, 1765-1845	O'Grady, T. et al.	2017
253	The impact of community-based interventions for the older population: a quasi-experimental study of a hip-fracture prevention program in Norway	Oien, H. et al.	2018
254	The tax-free year in Iceland: A natural experiment to explore the impact of a short-term increase in labor supply on the risk of heart attacks	Olafsdottir, T. et al.	2016
255	Effects of new motorway infrastructure on active travel in the local population: a retrospective repeat cross-sectional study in Glasgow, Scotland	Olsen, J. R. et al.	2016
256	Effect of a new motorway on social-spatial patterning of road traffic accidents: A retrospective longitudinal natural experimental study	Olsen, J. R. et al.	2017
257	Right-to-carry concealed weapon laws and homicide in large U.S. counties: The effect on weapon types, victim characteristics, and victim-offender relationships	Olson, D. E. et al.	2001
258	The impact of debt relief on under five mortality rate in Sub-Saharan Africa	Oryema, J. B. et al.	2017

259	A citywide smoking ban reduced maternal smoking and risk for preterm births: a Colorado natural experiment	Page, R. L, 2nd. et al.	2012
260	Co-Pay and Feel Okay: Self-Rated Health Status After a Health Insurance Reform	Paloyo, A. R.	2013
261	Does Education Improve Health? Evidence from Indonesia	Parinduri, R. A.	2017
262	To bike or not to bike? - Evidence from a university relocation	Peer, S.	2019
263	Cumulative receipt of an anti-poverty tax credit for families did not impact tobacco smoking among parents	Pega, F. et al.	2017
264	The implication of health insurance for child development and maternal nutrition: evidence from China	Peng, X. et al.	2016
265	Impact of the elimination of cost sharing for mammographic breast cancer screening among rural US women: A natural experiment	Peppercorn, J. et al.	2017
266	Access to Fluoridated Water and Adult Dental Caries: A Natural Experiment	Peres, M. A. et al.	2016
267	Evaluation of the impact of calorie labeling on McDonald's restaurant menus: a natural experiment	Petimar, J. et al.	2019
268	Carrots and sticks vs carrots: Comparing approaches to workplace travel plans using disincentives for driving and incentives for active travel	Petrunoff, N. et al.	2015
269	The Hound of the Baskervilles effect: natural experiment on the influence of psychological stress on timing of death	Phillips, D. P. et al.	2001
270	The costs and outcomes of restricting public access to poison control centers - Results from a natural experiment	Phillips, K. A. et al.	1998
271	The effect of the tobacco settlement and smoking bans on alcohol consumption	Picone, G. A. et al.	2004
272	Impact of a labour disruption affecting local public health on the incidence of chlamydia infections in Toronto	Pinto, A. D. et al.	2013
273	Conditional Cash Transfers, Food Security, and Health: Biocultural Insights for Poverty-Alleviation Policy from the Brazilian Amazon	Piperata, B. A. et al.	2016
274	Increasing powers to reject licences to sell alcohol: Impacts on availability, sales and behavioural outcomes from a novel natural experiment evaluation	Pliakas, T. et al.	2018
275	Getting shops to voluntarily stop selling cheap, strong beers and ciders: a time-series analysis evaluating impacts on alcohol availability and purchasing	Pliakas, T. et al.	2018
276	The persistent effects of minimum legal drinking age laws on drinking patterns later in life	Plunk, A. D. et al.	2013
277	The impact of public housing on social networks: a natural experiment	Pollack, C. E. et al.	2014
278	The effect of police intelligence on group violence: Evidence from reassignments in Sweden	Poutvaara, P. et al.	2009

279	Reduction in male suicide mortality following the 2006 Russian alcohol policy: an interrupted time series analysis	Pridemore, W. A. et al.	2013
280	Socioeconomic change and homicide in a transitional society	Pridemore, W. A. et al.	2016
281	Effects of urban motorways on physical activity and sedentary behaviour in local residents: a natural experimental study	Prins, R. G. et al.	2017
282	Causal pathways linking environmental change with health behaviour change: Natural experimental study of new transport infrastructure and cycling to work	Prins, R. G. et al.	2016
283	The effectiveness of a community playground intervention	Quigg, R. et al.	2012
284	Child bed net use before, during, and after a bed net distribution campaign in Bo, Sierra Leone	Ranasinghe, S. et al.	2015
285	Reductions in the United Kingdom's Government Housing Benefit and Symptoms of Depression in Low-Income Households	Reeves, A.et al.	2016
286	Introduction of a National Minimum Wage Reduced Depressive Symptoms in Low-Wage Workers: A Quasi-Natural Experiment in the UK	Reeves, A.et al.	2017
287	Impact of tobacco prices and smoke-free policy on smoking cessation, by gender and educational group: Spain, 1993-2012	Regidor, E. et al.	2015
288	The short-term impacts of Earned Income Tax Credit disbursement on health	Rehkopf, D. H. et al.	2014
289	The impact of the Ethiopian health extension program and health development army on maternal mortality: A synthetic control approach	Rieger, M. et al.	2019
290	The short-term impact of the alcohol act on alcohol-related deaths and hospital admissions in Scotland: a natural experiment	Robinson, M. et al.	2018
291	Evaluating the impact of the alcohol act on off-trade alcohol sales: a natural experiment in Scotland	Robinson, M. et al.	2014
292	A microenvironment approach to reducing sedentary time and increasing physical activity of children and adults at a playground	Roemmich, J. N. et al.	2014
293	Shall-issue policy and criminal activity among applicants for permits to carry concealed firearms	Romero, M. et al.	2003
294	Impact of the Cincinnati Aligning Forces for Quality Multi-Payer Patient Centered Medical Home Pilot on Health Care Quality, Utilization, and Costs	Rosenthal, M. B. et al.	2016
295	Pay for Performance in Medicaid: Evidence from Three Natural Experiments	Rosenthal, M. B. et al.	2016
296	The impact of small changes in bar closing hours on violence. The Norwegian experience from 18 cities	Rossow, I. et al.	2012
297	A food retail-based intervention on food security and consumption	Sadler, R. C. et al.	2013

298	Impact of the great recession on self-perceived health in Spain: a longitudinal study with individual data	Saez, M. et al.	2019
299	The Recreovia of Bogota, a Community-Based Physical Activity Program to Promote Physical Activity among Women: Baseline Results of the Natural Experiment Al Ritmo de las Comunidades	Sarmiento, O. L. et al.	2017
300	Effectiveness of a 2-year post-natal nurse home-visiting programme when children are aged 5 years: Results from a natural experiment	Sawyer, A. C. P. et al.	2019
301	Effectiveness of nurse home-visiting for disadvantaged families: results of a natural experiment	Sawyer, M. G. et al.	2013
302	The impact of telephonic wellness coaching on weight loss: A "Natural Experiments for Translation in Diabetes (NEXT-D)" study	Schmittdiel, J. A. et al.	2017
303	Effects of gentrification on health status after Hurricane Katrina	Schnake-Mahl, A. et al.	2020
304	Can food safety shortfalls disrupt 'Ag for Nutrition' gains? Evidence from Eid al-Adha	Schwab, B. et al.	2019
305	Is There an Association Between Gasoline Prices and Physical Activity? Evidence from American Time Use Data	Sen, B.	2012
306	The Impact of the Affordable Care Act's Dependent Coverage Mandate on Use of Dental Treatments and Preventive Services	Shane, D. M. et al.	2017
307	Pilot evaluation of HEAL - A natural experiment to promote obesity prevention behaviors among low-income pregnant women	Sharma, S. V. et al.	2018
308	Long-Term Associations Between Disaster Experiences and Cardiometabolic Risk: A Natural Experiment From the 2011 Great East Japan Earthquake and Tsunami	Shiba, K. et al.	2019
309	Effectiveness of a scaled up physical activity intervention in Brazil: A natural experiment	Simoes, E. J. et al.	2017
310	Primary birthing attendants and birth outcomes in remote Inuit communities-a natural "experiment'' in Nunavik, Canada	Simonet, F. et al.	2009
311	Change in Food and Beverage Availability and Marketing Following the Introduction of a Healthy Food Financing Initiative-Supported Supermarket	Singleton, C. R. et al.	2019
312	Did the suicide barrier work after all? Revisiting the Bloor Viaduct natural experiment and its impact on suicide rates in Toronto	Sinyor, M. et al.	2017
313	Alcohol and suicide in Denmark 1911-24--experiences from a 'natural experiment'	Skog, O. J.	1993
314	Changes in Alcohol-Problems as a Result of Changing Alcohol-Consumption - a Natural Experiment	Smart, R. G.	1987
315	On the sustainability of a family planning program in Nigeria when funding ends	Speizer, I. S. et al.	2019

316	The impact of food and nutrient-based standards on primary school children's lunch and total dietary intake: a natural experimental evaluation of government policy in England	Spence, S. et al.	2013
317	Maryland Alcohol Sales Tax and Sexually Transmitted Infections: A Natural Experiment	Staras, S. A. S. et al.	2016
318	Migration and mental health: Evidence from a natural experiment	Stillman, S. et al.	2009
319	The Daily Physical Activity (DPA) Policy in Ontario: Is lt Working? An Examination Using Accelerometry-measured Physical Activity Data	Stone, M. R. et al.	2012
320	Extra vitamin D from fortification and the risk of preeclampsia: The D-tect Study	Stougaard, M. et al.	2018
321	Effects of Prenatal Poverty on Infant Health: State Earned Income Tax Credits and Birth Weight	Strully, K. W. et al.	2010
322	How do changes to the built environment influence walking behaviors? A longitudinal study within a university campus in Hong Kong	Sun, G. et al.	2014
323	The spatio-temporal relationship between alcohol outlets and violence before and after privatization: A natural experiment, Seattle, Wa 2010-2013	Tabb, L. P. et al.	2016
324	Long-term follow-up on biological risk factors, adiposity, and cardiorespiratory fitness development in a physical education intervention: a natural experiment (CHAMPS-study DK)	Tarp, J. et al.	2018
325	The effect of increased alcohol availability on alcohol-related health problems up to the age of 42 among children exposed &ITin utero&IT: a natural experiment	Thern, E. et al.	2018
326	Effects of increased alcohol availability during adolescence on the risk of all-cause and cause-specific disability pension: a natural experiment	Thern, E. et al.	2017
327	No effects of increased alcohol availability during adolescence on alcohol-related morbidity and mortality during four decades: a natural experiment	Thern, E. et al.	2017
328	Is There an Increased Risk of Criminal Behavior Among Children Who Were In Utero When Their Mothers Were Exposed to Increased Alcohol Availability? A Register-Based Study Using a Natural Experiment Setting	Thern, E. et al.	2019
329	The impact of a new McDonald's restaurant on eating behaviours and perceptions of local residents: A natural experiment using repeated cross-sectional data	Thornton, L. E. et al.	2016
330	Disasters, donations, and tax law changes: Disentangling effects on subjective well-being by exploiting a natural experiment	Tiefenbach, T. et al.	2015
331	Changes in patterns of drug injection concurrent with a sustained reduction in the availability of heroin in Australia	Topp, L. et al.	2003
332	Assessing the effect of physical activity classes in public spaces on leisure-time physical activity: "Al Ritmo de las Comunidades" A natural experiment in Bogota, Colombia	Torres, A. et al.	2017

333	Effects of a multi-component responsible beverage service programme on violent assaults in Sweden	Trolldal, B. et al.	2013
334	The causal effect of education on HIV stigma in Uganda: Evidence from a natural experiment	Tsai, A. et al.	2015
335	Early-Life Nutritional Programming of Type 2 Diabetes: Experimental and Quasi-Experimental Evidence	Vaiserman, A. et al.	2017
336	Competitive Food Sales in Schools and Childhood Obesity: A Longitudinal Study	Van Hook, J. et al.	2012
337	Effects of changes to the taxation of beer on alcohol consumption and government revenue in Australia	Vandenberg, B. et al.	2019
338	Do alcohol restrictions reduce suicide mortality?	Varnik, A. et al.	2007
339	Park improvements and park activity: a natural experiment	Veitch, J. et al.	2012
340	The REVAMP natural experiment study: the impact of a play-scape installation on park visitation and park-based physical activity	Veitch, J. et al.	2018
341	Benefits of smoking bans on preterm and early-term births: a natural experimental design in Switzerland	Vicedo-Cabrera, A. M. et al.	2016
342	Cancer incidence following long-term consumption of drinking water with high inorganic selenium content	Vinceti, M. et al.	2018
343	A Change from Public to Private Sale of Wine - Results from Natural Experiments in Iowa and West-Virginia	Wagenaar, A. C. et al.	1991
344	The individual and program impacts of eliminating Medicaid dental benefits in the Oregon Health Plan	Wallace, N. T. et al.	2011
345	Choice architecture modifies fruit and vegetable purchasing in a university campus grocery store: time series modelling of a natural experiment	Walmsley, R. et al.	2018
346	Mode of HPV vaccination delivery and equity in vaccine uptake: A nationwide cohort study	Wang, J. et al.	2019
347	The effect of environmental regulation on air quality: A study of new ambient air quality standards in China	Wang, K. L. et al.	2019
348	Health care utilization and health outcomes: a population study of Taiwan	Wang, S-Y. et al.	2012
349	Economic Insecurity and the Weight Gain of Canadian Adults: A Natural Experiment Approach	Watson, B. et al.	2016
350	Changes in children's sleep and physical activity during a 1-week versus a 3-week break from school: a natural experiment	Weaver, R. G. et al.	2019
351	Impact of China's essential medicines scheme and zero-mark-up policy on antibiotic prescriptions in county hospitals: a mixed methods study	Wei, X. et al.	2017

352	The effect of medical marijuana laws on adolescent and adult use of marijuana, alcohol, and other substances	Wen, H. et al.	2015
353	Improving Mental Health Through the Regeneration of Deprived Neighborhoods: A Natural Experiment	White, J. et al.	2017
354	Hospital admission rates for alcoholic intoxication after policy changes in the canton of Geneva, Switzerland	Wicki, M. et al.	2011
355	Public smoking bans and self-assessed health: Evidence from Great Britain	Wildman, J. et al.	2012
356	Population impact of reimbursement for smoking cessation: a natural experiment in The Netherlands	Willemsen, M. C. et al.	2013
357	Does liberalizing cannabis laws increase cannabis use?	Williams, J. et al.	2014
358	The Income and Health Effects of Tribal Casino Gaming on American Indians	Wolfe, B. et al.	2012
359	Health returns to modern heating: evidence from China	Xue, Y. H.	2018
360	Estimated increase in cross-border purchases by Washington residents following liquor privatization and implications for alcohol consumption trends	Ye, Y. et al.	2016
361	Diesel vehicle emission and death rates in Tokyo, Japan: a natural experiment	Yorifuji, T. et al.	2011
362	Maternal benzene exposure and low birth weight risk in the United States: a natural experiment in gasoline reformulation	Zahran, S. et al.	2012
363	Is a reduction in distance to nearest supermarket associated with BMI change among type 2 diabetes patients?	Zhang, Y. T. et al.	2016
364	High quit ratio among Asian immigrants in California: implications for population tobacco cessation	Zhu, S-H. et al.	2007
365	Does Daylight Savings Time encourage physical activity?	Zick, C. D.	2014
366	Long-range fine particulate matter from the 2002 Quebec forest fires and daily mortality in Greater Boston and New York City	Zu, K. et al.	2016

Table S3. As-if randomisation validation exercise

Rater #	1	2	3	4	5	6	7	8	10	11	12
NEE study	Probability of as-if randomisation										
1 (Aittasalo)	Possibly	Possibly	Possibly	Likely	Unsure or Cannot tell	Unsure or Cannot tell	Likely	Likely	Implausible	Possibly	Possibly
2 (Baltagi)	Possibly	Likely	Probably	Likely	Likely	Implausible	Likely	Probably	Likely	Likely	Likely
3 (Behrman)	Possibly	Implausible	Probably	NA	Likely	Likely	Likely	Probably	Likely	Likely	Likely
4 (Casey)	Possibly	Implausible	Possibly	NA	Likely	Probably	Probably	Probably	Implausible	Probably	Probably
5 (Dai)	Possibly	Probably	Likely	NA	Unsure or Cannot tell	Unsure or Cannot tell	Possibly	Possibly	Implausible	Probably	Probably
6 (Dudovitz)	Implausible	Likely	Likely	NA	Likely	Likely	Likely	Likely	Likely	Likely	Likely
7 (Dursun)		Probably	Probably	Probably	Likely	Possibly	Likely	Probably	Probably	Possibly	Possibly
8 (Egan)	Unsure or Cannot tell	Possibly	Likely	Possibly	Implausible	Implausible	Implausible	Likely	Implausible	Possibly	Implausible
9 (Evans)	Unsure or Cannot tell	Unsure or Cannot tell	Unsure or Cannot tell	NA	Implausible		Possibly	Unsure or Cannot tell	Likely	Implausible	Unsure or Cannot tell
10 (Goodman)	Implausible	Probably	Likely	Implausible	Likely	Probably	Likely	Likely	Likely	Probably	Probably
11 (Grabovac)	Possibly	Probably	Possibly	NA	Likely	Implausible	Possibly	Possibly	Likely	Likely	Probably
12 (Gunes)	Implausible	Possibly	Probably	Likely	Likely	Likely	Likely	Probably	Likely	Possibly	Likely
13 (Huang)	Implausible	Unsure or Cannot tell	Implausible	NA	Likely	Possibly	Unsure or Cannot tell	Possibly	Likely	Possibly	Unsure or Cannot tell
14 (Janszky)	Possibly	Possibly	Likely	NA	Likely	Likely	Likely	Possibly	Likely	Likely	Possibly
15 (Koh)	Possibly	Implausible	Probably	NA	Implausible	Implausible	Possibly	Possibly	Unsure or Cannot tell	Likely	Possibly
16 (McIsaac)	Possibly	Implausible	Possibly	NA	Implausible	Implausible	Implausible	Possibly	Implausible	Implausible	Implausible
17 (Milicic)	Probably	Probably	Possibly	NA	Implausible	Likely	Possibly	Possibly	Implausible	Possibly	Probably
18 (Poutvaara)	Possibly	Probably	Probably	NA	Likely	Likely	Probably	Possibly	Likely	Implausible	Probably
19 (Smart)	Implausible	Unsure or Cannot tell	Implausible	NA	Implausible	Implausible	Implausible	Unsure or Cannot tell	Implausible	Implausible	Possibly
20 (Veitch)	Implausible	Possibly	Possibly	NA	Implausible	Implausible	Implausible	Probably	Implausible	Implausible	Implausible
